# The FcRn from gene to protein and function: comparison between species

**DOI:** 10.3389/fimmu.2025.1608426

**Published:** 2025-08-01

**Authors:** Luz Belinda Ortiz-Alegría, Lizbeth Xicoténcatl-García, Irma Cañedo-Solares, Claudia Patricia Rico-Torres, Fernando Gómez-Chávez

**Affiliations:** ^1^ Laboratorio de Inmunología Experimental, Instituto Nacional de Pediatría, Secretaría de Salud, Mexico City, Mexico; ^2^ Laboratorio de Enfermedades Osteoarticulares e Inmunológicas, Escuela Nacional de Medicina y Homeopatía, Instituto Politécnico Nacional, Mexico City, Mexico

**Keywords:** FcRn, species, IgG, albumin, phylogeny, ontogeny

## Abstract

Immunoglobulin Fc receptors are crucial molecules in immunological processes that help maintain homeostasis following internal or external stimuli. Most of the specific gamma recognition molecules (FcγRI, II, and III) are known for their roles in phagocytosis and cellular cytotoxicity against various pathogens and transformed cells and in regulating the humoral immune response. Within this family of IgG Fc receptors, there is a structurally similar receptor with different functions: the so-called neonatal receptor for the Fc fraction of IgG, or FcRn, which is primarily associated with IgG and albumin homeostasis, the transfer of immunity from mother to offspring, and the regulation of the immune response in mammals. Therefore, this molecule could be considered “the regulator and transporter of the main blood proteins” from the blood vessels and the lumen of the mucosa to the tissues of the newborn and neonate, through the epithelium and endothelium. It may act as a trans-tissue and interindividual “protector,” as it mediates the transfer of IgG antibodies to the sites where they are needed. Additionally, it regulates plasma albumin and IgG concentrations, contributing to the balance of body fluids. Although there is abundant literature on this receptor, some phenomena remain unexplored or poorly understood. In particular, the variations in its functions across different cell types and between species, how they influence IgG and albumin levels in various body fluids, and the pathways involved in immunity transmission need further investigation. In this paper, we present a comprehensive review of original research articles and analyses focused on the gene, mRNA, and protein composition of the FcRn, with the aim to compare the genetic, structural, and functional characteristics in different mammalian species, focusing on its role in immunity and homeostasis, as well as the ontogeny and phylogeny of the FcRn.

## 1 Introduction

The neonatal Fc receptor (FcRn) was identified through the efforts of several groups. Its conceptual basis dates back to 1880, when Von Behring and Kitasato discovered “products” in guinea pig serum that neutralized *Corynebacterium diphtheriae* and conferred protective immunity (1). They also demonstrated that passive immunization with serum from immunized animals contained “antitoxins,” corresponding to what are now called antibodies, which could effectively prevent or treat infection. Ehrlich (1892) and Wernicke (1895) demonstrated that female guinea pigs, mice, and rabbits immunized against diphtheria produced offspring resistant to the disease and that the intensity of the maternal immune response determined the level of passive immunity transmitted to the offspring (2, 3). Studies in mice reported maternal lactation (postnatal transmission), even from wet nurses, was critical in transferring immunity against infectious agents. These data laid the foundations for passive maternal immunity, which has been confirmed in numerous mammalian species (3–5). Francis Brambell and his colleagues conducted several studies and were the first to describe the transfer of maternal plasma proteins to the yolk sac cavity of rabbits (6). These proteins corresponded to antibodies transferred from the second half of pregnancy onwards, increasing at the end. In rabbits, the ability to “accept” maternal antibodies was identified as variable in their offspring (6–8), and transfer during lactation occurred through the offspring’s intestines, specifically in the jejunum and ileum (8, 9). This passage of antibodies was selective, and heterologous or nonspecific sera blocked it, suggesting the presence of a specific receptor at that time (7, 10, 11). Other studies indicated that antibody transmission occurred by pinocytosis of the yolk sac border in rabbit fetuses (12) and the intestine of neonatal rats (13).

Furthermore, they noted that there are variations in the transfer of passive immunity among species. In species like humans, primates, rabbits, and guinea pigs, IgG antibodies are transferred during gestation, reaching levels in the newborn that are equal to or higher than those in the mother, and increasing with breastfeeding. In contrast, in cows, goats, sheep, horses, donkeys, and pigs, immunity is transmitted only from colostrum to the newborn’s circulation through the intestine. This ability to cross the intestinal epithelium is transient and lasts a few hours. Species such as mice, rats, hedgehogs, cats, and dogs present a mixed passive immunity: part of the immunity is transmitted during gestation, but most is given through breastfeeding and ceases abruptly around three weeks (14–17). Regarding another function of FcRn, protecting IgG from catabolism, Brambell et al. (1966) proposed the existence of a specific receptor, and they described a relationship between its plasma concentration and its fractional catabolism rate (18). All these data together led Brambell and collaborators to propose the existence of two receptors capable of binding IgG, each one developing one of the described functions. As was later shown, they corresponded to the same receptor, the FcRn. The contemporaries of Brambell, Schultze and Heremans (1966) suggested that the mechanism of protection against IgG degradation in blood was also applicable to albumin. Nowadays, it has been shown that FcRn also binds to albumin and carries out different functions, like those described for IgG, which will be seen later. Hence, both molecules have unusually long half-lives -almost three weeks in humans- compared to other plasma proteins; both are distinguished from all other soluble proteins by a direct relationship between their plasma concentrations and fractional catabolic rates, considering the mass fraction of protein catabolized in plasma per unit of time (18).

## 2 FcRn gene

### 2.1 Gene structure

**Figure 1:**
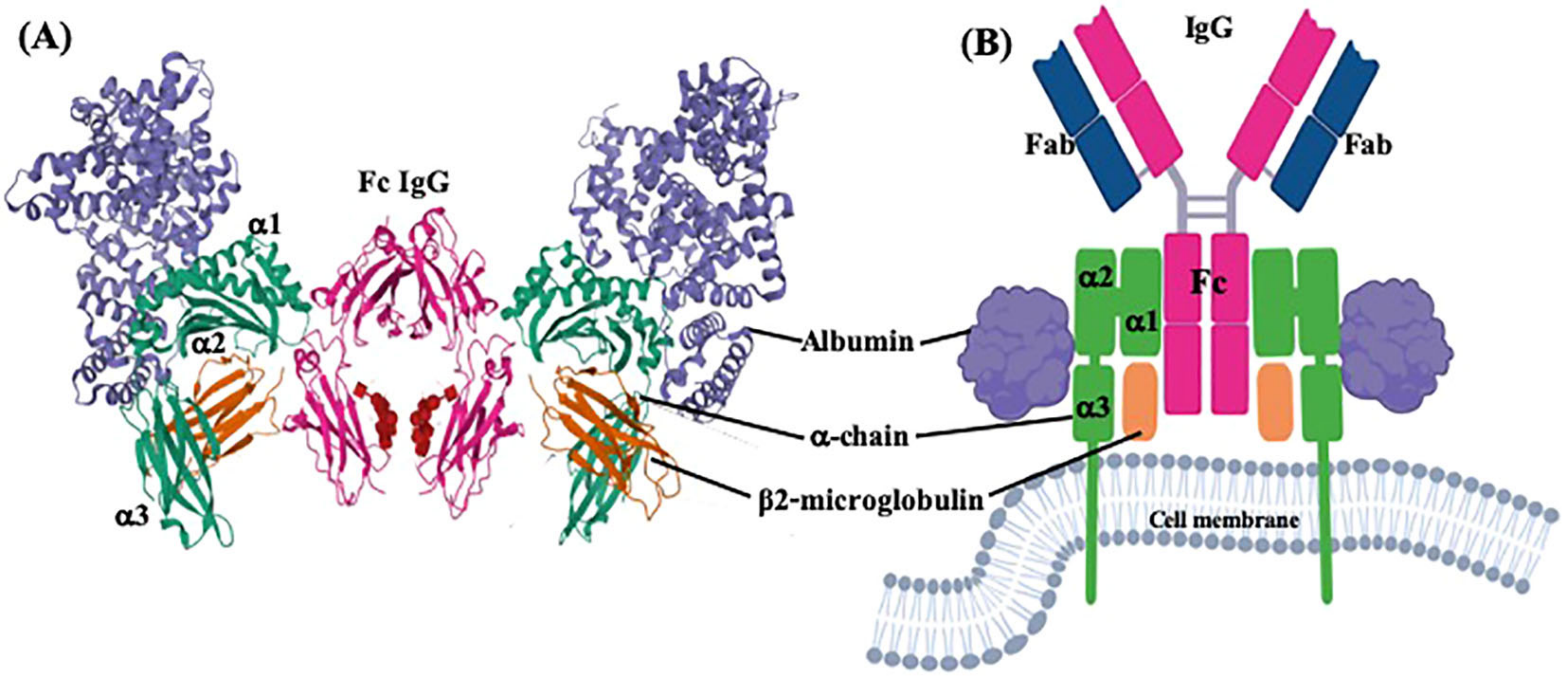
Three dimensional and plain structure models of FcRn bound to IgG and albumin. Two FcRn molecules bound to one IgG monomer and two albumin molecules. **(A)** Ternary complex between FcRn, albumin and Fc of IgG from PDB ID: 4N0U, DOI: 10.1074/jbc.M113.537563, and **(B)** molecular model created with BioRender.com.

**Figure 2:**
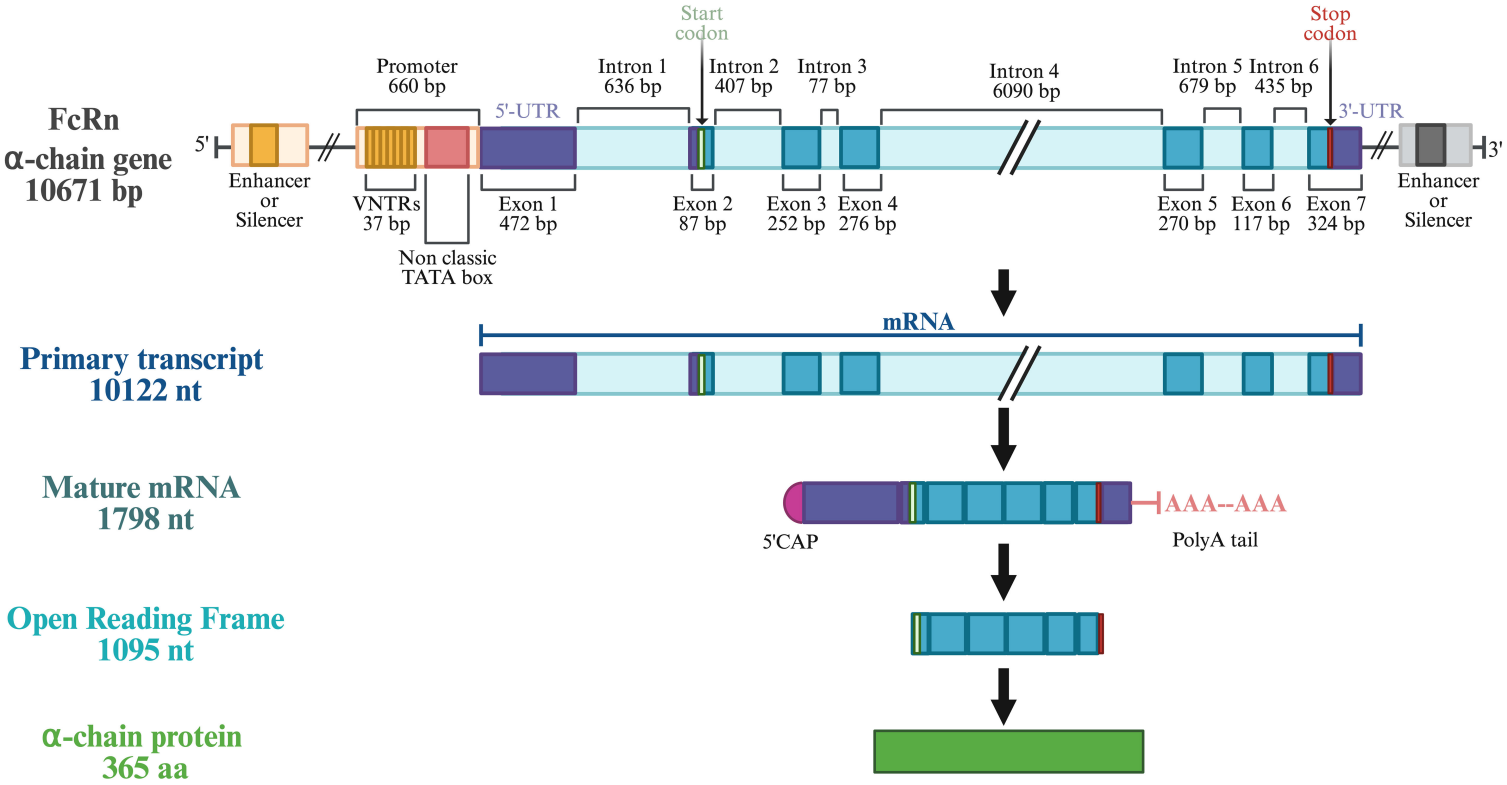
General structure of human FcRn α-chain gene (*FCGRT*). Modified with BioRender.com from Mikulska et al, 2000; Mikulska and Simister, 2000.

The FcRn consists of β2-microglobulin and an α-chain (**Figure 1**), encoded by the *B2M* and *FCGRT* genes, respectively (19, 20). *B2M* is found on chromosome 15 with no known genetic variants (20, 21). Variations in FcRn expression stem mainly from the α-chain. The *FCGRT* gene is located on chromosome 19, sequenced in 2000, and is structured similarly to MHC α-chain genes, with six introns and seven exons. It spans about 10,671 bp, with a transcribed region of 1798 bp and a 1095 bp coding region, producing a protein of 365 amino acids (**Figure 2** and **Supplementary Table 1**) (20, 22–26). The gene features a 660 bp downstream promoter with two initiation sites and a non-classical TATA box, lacking typical active component features. The 5’-UTR includes parts of exons 1 and 2, while the 3’-UTR covers part of exon 7 (**Figure 2**) (23, 24).

Variations in the intron/exon structure of the FcRn gene across species (**Supplementary Table 1**) could have significant implications for its function, expression, and role in immune responses. For instance, the length of the cytoplasmic tail, encoded in exon 6 of the FcRn α-chain, varies between species, with animals such as cows and pigs having shorter tails compared to humans and rodents (23). Although this variation does not affect IgG transport *in vivo*, it may influence polarized IgG secretion *in vitro* (27). Additionally, polymorphisms within the FcRn gene have functional consequences. In pigs, a single-nucleotide polymorphism (SNP) in exon 6 is associated with serum IgG levels, highlighting the potential for genetic variation to modulate immune responses (28).

Recently, four new splice variants have been discovered in mice and humans, located in specific vesicles where immunoglobulins and albumin are stored. Understanding these splice variants could reveal additional layers of regulation in IgG transport and stability, underscoring the complexity of FcRn gene function across species (29). Further research is needed to elucidate the functional significance of these splice variants in immune modulation. Similarly, a splicing variant has been identified in pigs that encodes a truncated protein, which is predominantly retained in the lysosomal compartment and is not found on the cell surface, lacking a portion of exon two involved in IgG binding, maybe working as a regulatory variant or an intermediate rapidly degraded (30). While the overall structure of the FcRn gene is conserved, species-specific variations in intron length and exon usage may contribute to differences in FcRn function and IgG handling between species.

### 2.2 Gene similarities/dissimilarities among species

The human *FCGRT* has orthologous genes; there are between 240 and 250 orthologous sequences from mammals, some from birds and reptiles (59 species), and some from fish (66 species), whose nucleotide sequences are reported in the Ensemble and NCBI databases^1, 2^ some are still putative, and others have already been demonstrated and validated by different methods. In the Ensemble database, information about *FCGRT* in 89 species was found, including humans; 342 transcripts are described, some with alternative splicing variants. In this review, we consider the sequences of the most common domestic animals and some wild animals, which have a phylogenetically close relationship with humans, and others, as will be seen later, with different types of mother-child passive immunity transfer to analyze the influence of FcRn in this phenomenon (**Supplementary Table 1**). Moreover, there are multiple ways to classify the placentation of species. For this work, the first choice was to classify them according to their histological characteristics and subsequently by the distribution of the chorionic villi. At least one species was chosen per order and type of placenta, whose database sequence was complete or validated for bioinformatics analysis.

The location and characteristic features of the α-chain gene of twenty mammalian species are described in **Supplementary Table 1**, which is known in most species, except in rabbits and bushbabies, according to the Ensemble and Uniprot databases. The *FCGRT* exhibits variation in length and number of introns/exons among species and even among strains of the same species, such as in mice, dogs, and pigs, although most have between 6 and 7 exons. The distribution and sizes of exons and introns are consistent among species within the same order. The species were further classified based on their type of placentation (see below for details of the classification). This classification is pertinent as it influences the distribution of FcRn in various organs and tissues during gestation. As we will explore later, this distribution pattern directly affects the transfer of immunity, mainly through transporting maternal antibodies to fetuses or newborns. As observed, the whale and the pig have the longest *FCGRT* genes, with 9 and 11 exons, respectively, and significant variability in their size between pig breeds (**Supplementary Table 1**). The length of mRNA also exhibits variations; nonetheless, the size and structure of the polypeptide α-chain remain conserved at approximately 365 amino acids, as shown in **Supplementary Table 1**.

**Figure 3:**
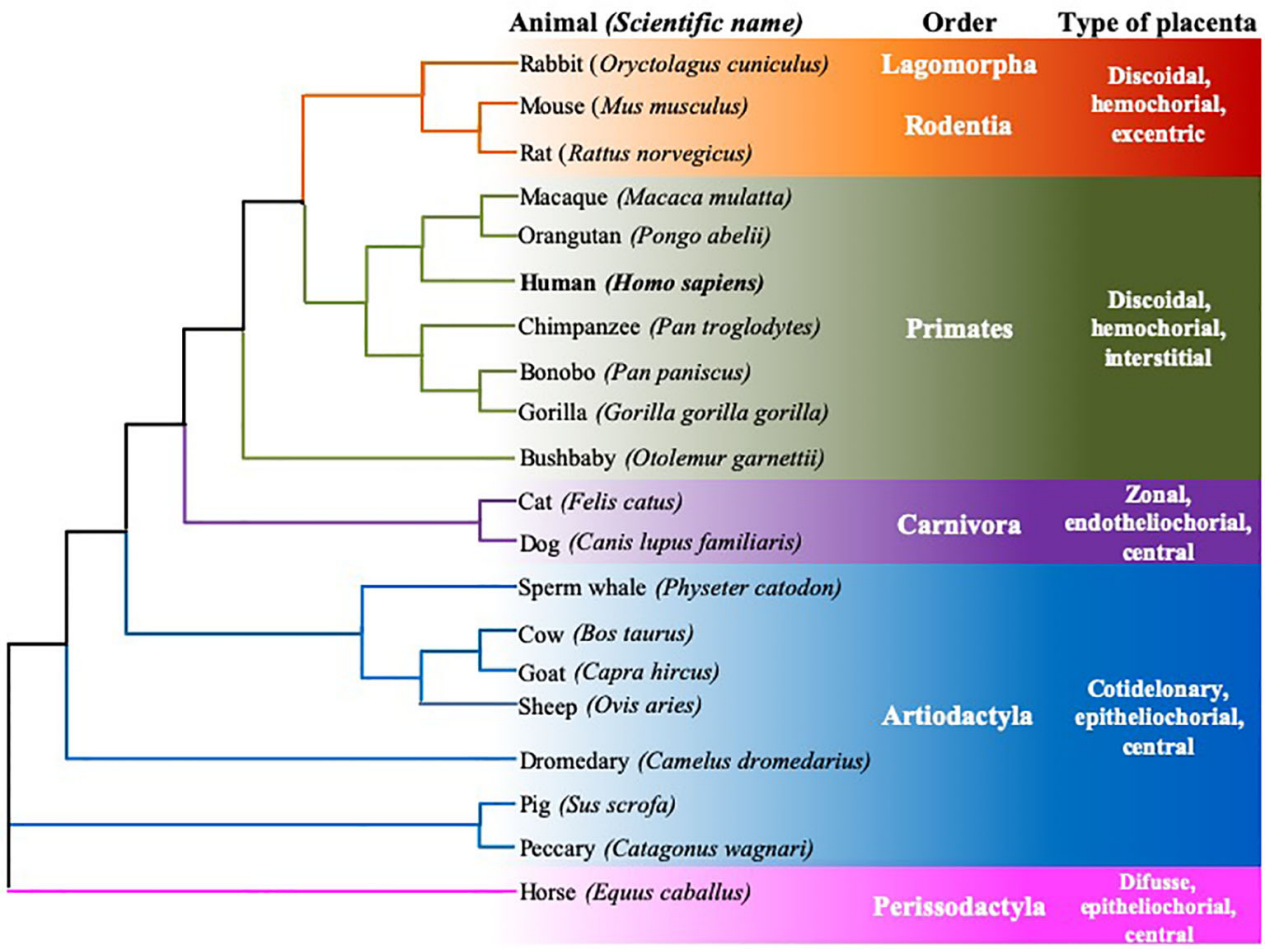
Rooted neighbor joining tree showing the phylogeny of FcRn α-chain mRNA sequences of twenty mammalian species. Six animal orders are considered, species are grouped according to their type of placenta, using the data of Ensamble and the EMBL-EBI Clustal Omega program.

**Figure 4:**
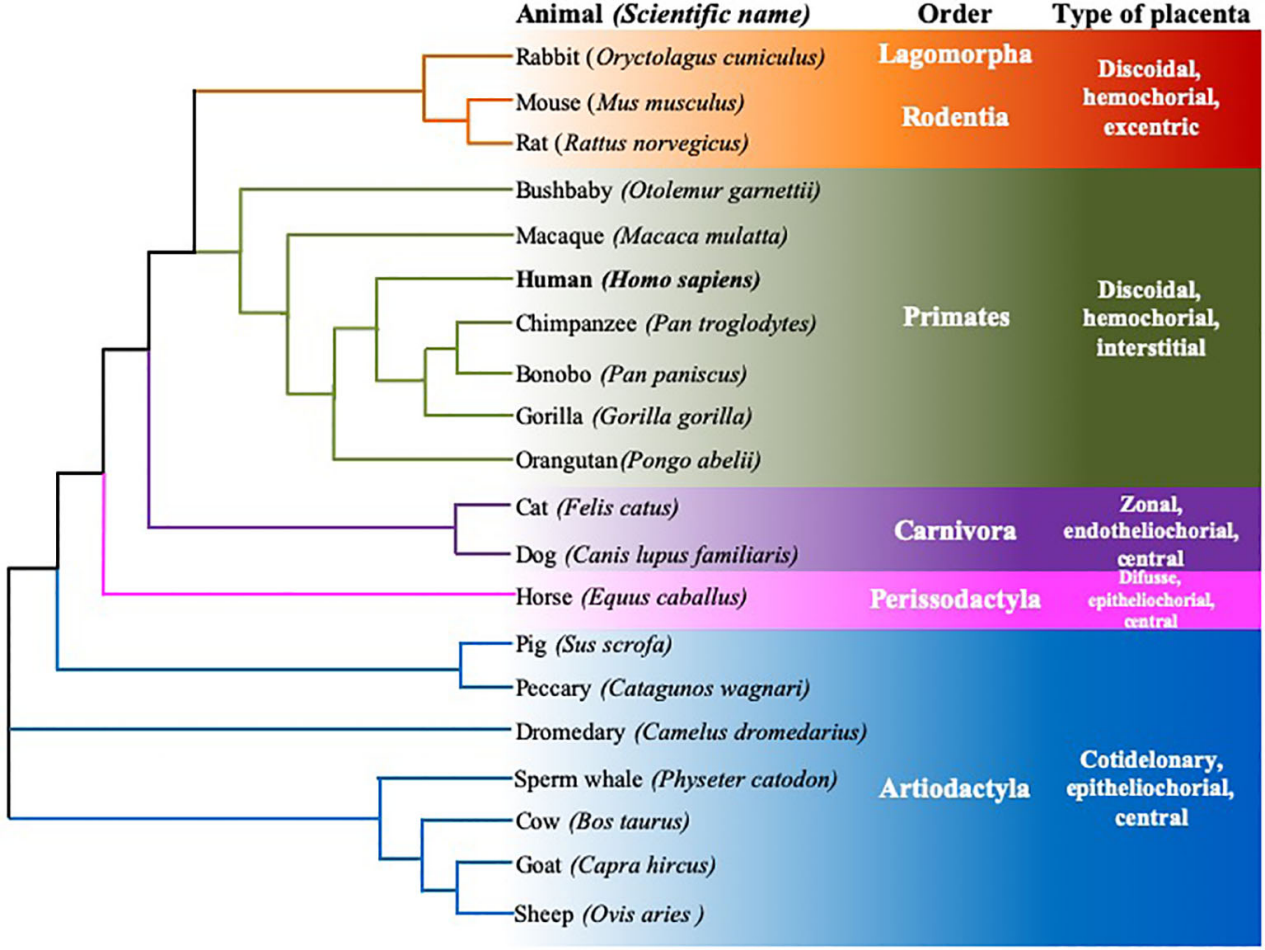
Rooted neighbor joining tree showing the phylogeny of FcRn α-chain protein sequences of twenty mammalian species. Six animal orders are shown, species are grouped according to their type of placenta, using the data of Ensamble and the EMBL-EBI Clustal Omega program.

Furthermore, both the size of the signal peptide and the α domains are consistent. Additionally, the protein’s expression across different cell types and tissues in various species and its functions appear to be conserved. The comparison of cDNA and amino acid sequences supports this observation. Sequences were obtained from the Ensemble^1^ and UniProt^2^ ^,^ ^3^ databases, and subsequent analysis confirms this consistency. The accession numbers of the considered sequences are found in the **Appendix** section. Although there are differences in the disposition of exons/introns, the homology between the cDNA sequences is the largest among species of the same Order (**Figure 3**). This species classification, in terms of the order to which they belong and the type of placenta, was also used to compare the homology of genes, mRNA, and protein sequences (**Figures 3**, **4**).

### 2.3 Gene expression and its regulation

**Figure 5:**
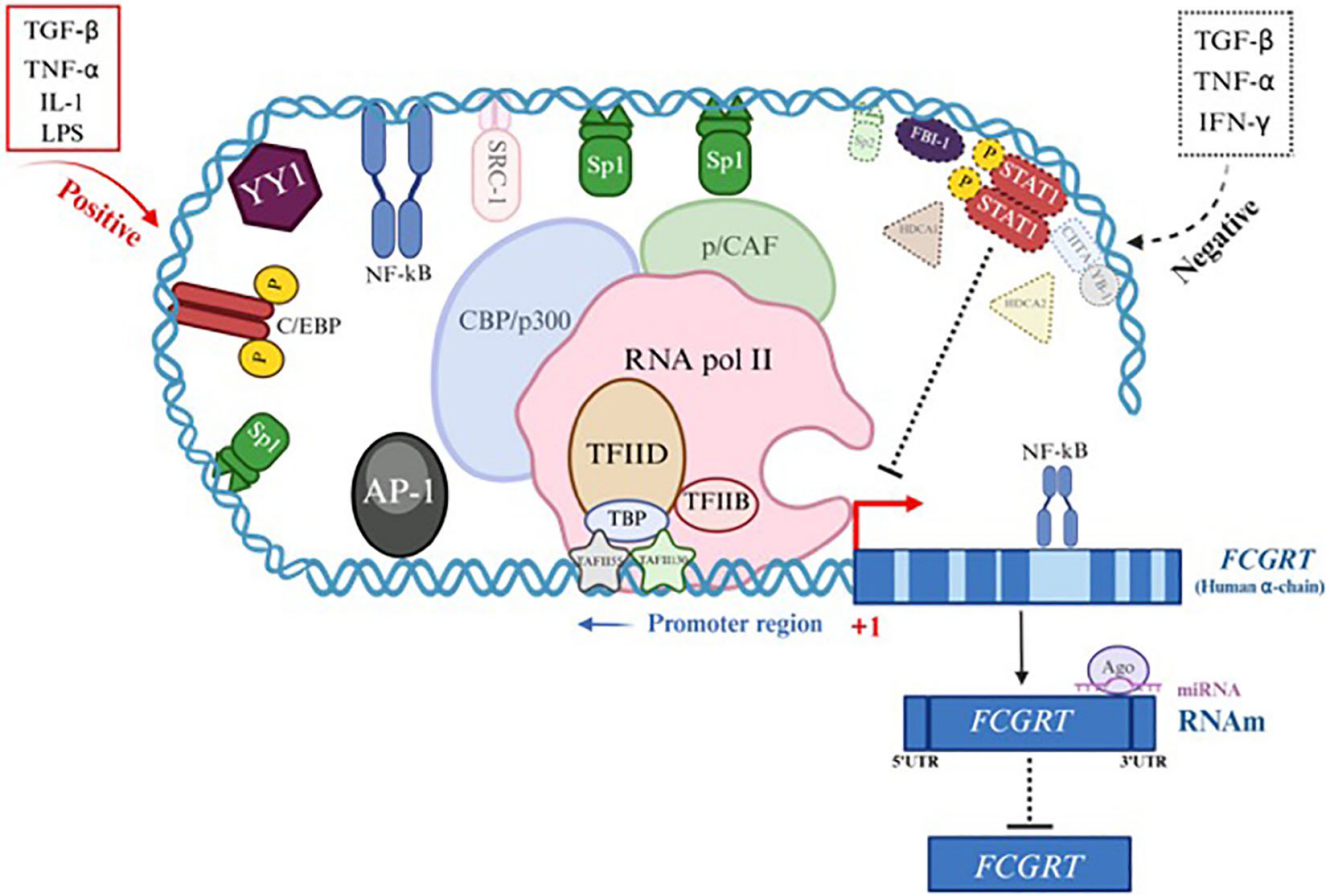
Gene expression and regulation of FcRn α-chain gene (*FCGRT*). Transcriptional or post-transcriptional regulation of the *FCGRT* gene suggests the presence of collaborative interactions between specific transcription factors (TF) binding sites in the promoter region of the gene to form a dynamic and complex protein machinery that interact with each other to initiate activation or inactivation of the *FCGRT* α-chain promoter depending on the cytokine involved and cell type (See **Supplementary Table 2**). Examples of Positive regulation (red) involves different TFs (line and solid color) and coactivators (line and faint color). Negative regulation (dotted black) recruits other TFs (dotted line and continuous color) and corepressors (dotted line and faint color).

The exact mechanisms involved in *FCGRT* expression remain to be revealed; however, several data have implicated cytokines and specific Microorganism-Associated Molecular Patterns (MAMPs) as significant regulators of its transcription in various cell types in humans and mice, potentially triggering a dynamic protein interaction complex on the *FCGRT* promoter. Considering data from other similar genes and the *FCGRT* sequence, an effort has been made to establish associated mechanisms, including activators and repressors, as detailed in **Figure 5**; **Supplementary Table 2** (23, 24, 27–103). Evidence suggests that both pro-inflammatory and anti-inflammatory environments could regulate *FCGRT* transcription. For a long time, the prevailing paradigm assumed that pro-inflammatory stimuli could upregulate, while anti-inflammatory molecules would inhibit the expression of this molecule (27). However, there is contradictory data, and for instance, some reports have implicated anti-inflammatory cytokines such as TGF-β in stimulating *FCGRT* expression, which has been linked to food allergen tolerance in neonatal mice (27).

Furthermore, TNF-α, a pro-inflammatory cytokine, mediates various signaling pathways, including the NF-κB pathway, which is involved in the transcriptional regulation of numerous genes. FcRn expression in human retinal pigment epithelium (RPE) cells was downregulated following TNF-α stimulation, suggesting that NF-κB may negatively regulate it by interacting with transcriptional corepressors such as AP-1 and by recruiting histone deacetylase complexes (HDACs), which can directly interact with the p65 subunit of NF-κB and likely exert their corepressor function through these interactions (74, 88). Additionally, in an *in vitro* study using thyrocyte monolayers stimulated with IFN-γ and TNF-α before IgG contact, a significant inhibition of basolateral to apical IgG transport was observed when compared to untreated groups, indicating an effect of both cytokines on FcRn function and potentially on expression (104). Although this gene promoter lacks a typical TATA box and an Inr element, it does present specific binding sites for transcription factors (TFs), such as those from the SP family (SP1, Sp2, and Sp3), AP1 (c-Fos/c-Jun), the YY1 family (Yin-Yang 1), C/EBP, and NF-κB, which can bind to it in the -660 to -233 region. Once activated, these TFs can engage with an intricate network of coactivators or repressors, modulating *FCGRT* transcription. This phenomenon appears to be organism-, cell-type-, and environmental-stimulus dependent. Some data presented here derive from *in vitro* studies using transformed cells, as they do not necessarily mimic physiological FcRn expression in normal cells, even if they share the exact origin (**Supplementary Table 2**) (23, 24, 32–34, 39). Interestingly, intron-mediated transcriptional regulation has been observed in the *FCGRT* gene. NF-κB binding sites have been localized within intronic regions, primarily in the longer ones, suggesting that NF-κB may interact via loop interactions with the promoter and cooperate with other transcriptional elements such as the Sp1 family, AP1, and C/EBP, which are often present or act synergistically in NF-κB-regulated genes to achieve maximal promoter activation during transcription initiation in response to different stimuli such as TNF-α, IL-1, or LPS (67). The p300/CBP coactivator proteins possess histone acetyltransferase (HAT) activity, which neutralizes the positive charge of histones, reduces their affinity for DNA, and facilitates access to the promoter to activate transcription (51). However, p300/CBP is involved in a negative transcriptional regulation of *FCGRT* (85). After the interaction of IFN-R with IFN-γ, JAK1 and JAK2 (Janus Tyrosine Kinase) are activated and subsequently phosphorylate STAT-1 (Signal Transducer and Activator of Transcription-1), which dimerizes, translocates to the nucleus, and binds to β-activated sequences (GAS). This dimer may sequester CBP/p300, thereby preventing access to the promoter by other regulatory elements (85).

As described, the transcriptional or post-transcriptional regulation of *FCGRT* suggests collaborative interactions among specific transcription factor (TF) binding sites in the gene promoter region, forming a large, dynamic, and complex protein machinery that interacts with one another to initiate promoter activation or inactivation, depending on the cytokine involved and the cell type (**Supplementary Table 2**). **Figure 5** presents a scheme that illustrates the probable mechanisms of positive regulation (red) involving different TFs (solid line and color) and coactivators (dark line and color). Negative regulation (dashed black) recruits other TFs (dotted line and solid color) and corepressors (dashed line and dark color). These processes only approximate what occurs at the human gene level; therefore, several studies are necessary to corroborate the exact data. In other species, the mechanisms involved are also uncertain; however, it can be assumed that they are similar to those described here.

### 2.4 Polymorphisms

Limited data on the effects of *FCGRT* polymorphism on expression or function are available. Some studies have identified polymorphisms in the FcRn α-chain gene in humans and only a few in mammalian species (**Table 1**) (105–107). In humans, direct sequencing analyses of PCR products isolated from ten placental mRNAs of Canadian patients revealed the presence of two synonymous single nucleotide polymorphisms (SNPs), substitutions G251T and C707T, which correspond to the amino acids Pro19 and Arg171 of the mature polypeptide, with no effect on expression levels or receptor activity; however, the number of analyzed alleles was low (n=20) (108). In Japanese individuals, the *FCGRT* 5’ region and the exons of 126 subjects were sequenced, finding 33 genetic variants, among which two non-synonymous and heterozygous SNPs G629A (R210Q) and T889A (S297T) were analyzed in HeLa cells, showing no functional significance; both variants displayed similar intracellular localization and antibody recycling efficiencies (109).

**Table 1:** VNTRs of the human FcRn α-chain promoter and their association with some characteristics.

Country	Characteristics of the individuals (n)	Allele frequency (%)	Association	Reference
Germany	Blood donors (447)	VNTR1: 0.1	Higher transcriptional activity for VNTR3 than VNTR2. Monocytes from VNTR3/3 homozygous expressed 1.66 times more FcRn mRNA than VNTR2/3 and also bound more polyvalent IgG	(116)
		VNTR2: 7.5		
		VNTR3: 92.0		
		VNTR4: 0.2		
		VNTR 5: 0.2		
China	Lupus nephritis patients (200) and healthy (204)	VNTR1: 0.0	There was no association of the genotype with lupus nephritis, nor with indicators of renal, pathological and clinical prognosis. There was also no association with serum levels of autoantibodies	(118)
		VNTR2: 3.9		
		VNTR3: 95.9		
		VNTR4: 0.3		
		VNTR5: 0.0		
Czech Republic	Newborns (206) and mother/child pairs (103)	VNTR1: 0.2	There was no association with total levels of circulating IgG, nor with IgG subclasses, neither in mothers nor in newborns	(120)
		VNTR2: 6.4		
		VNTR3: 92.6		
		VNTR4: 0.7		
		VNTR5: 0.0		
Czech Republic	Common variable immunodeficiency (CVID) patients (62)	VNTR1: 0.0	There were no differences between VNTR3/3 homozygous and VNTR2 allele carriers in clinical or laboratory features, respiratory tract infections, lung structure and functional abnormalities, or other CVID phenotypic features	(119)
		VNTR2: 8.9		
		VNTR3: 90.3		
		VNTR4: 0.8		
		VNTR5: 0.0		
Japan	Cancer patients (126)	VNTR2: 3.2	----	(115)
		VNTR3: 96.8		
France	CVID patients (275) treated with IVIg and SCIG		VNTR3/3 patients had better efficiency of IgG replacement therapy. IVIg-treated patients with unusual genotype had lower IgG levels and efficiency than VNTR3/3. There were no differences in albumin levels and there was no association with the clinical phenotype	(121)
		VNTR2: 10		
		VNTR3: 89		


England	CVID patients (105) treated with IVIg and SCIG		VNTR3/3 patients had better efficiency of IgG replacement therapy. IVIg-treated patients with unusual genotype had lower IgG levels and efficiency than VNTR3/3. There were no differences in albumin levels and there was no association with the clinical phenotype	(121)
		VNTR2: 9		
		VNTR3: 90		


France	Metastatic colorectal cancer patients treated with cetuximab (94) and healthy individuals (198)	VNTR1: 0.0	VNTR3 homozygous patients had a lower distribution of cetuximab than VNTR2/3 and VNTR3/4. Genotype do not affect elimination. VNTRs influence the distribution of mAbs in the organism	(122)
		VNTR2: 8.4		
		VNTR3: 90.4		
		VNTR4: 1.0		
		VNTR5: 0.2		
France	Common variable immunodeficiency (CVID) patients (302) and controls (202)		Less common genotypes had lower IgG levels and efficiency. There was no difference in serum albumin levels. VNTR3/3 with a single infection had the highest IgG efficiency. There was no correlation between the genotype and the CVID phenotype	(123)
		VNTR2: 8.9, 7.9		
		VNTR3: 88.4, 88.1		


Holland	Multifocal motor neuropathy patients treated with IVIg (29)		There was no association with IgG levels or response to treatment	(113)
		VNTR2: 5.5		
		VNTR3: 88.9		
		VNTR4: 2.8		
		VNTR5: 2.8		
Spain and Greece	Rheumatoid arthritis patients treated with TNF inhibitors: Infliximab (IFX), etanercept (ETC) or adalimumab (ADM) (423)		There was no association with response to TNF inhibitors	(111)
		VNTR2: 11.9		
		VNTR3: 87.2		
		VNTR4: <1.0		
		VNTR5: <1.0		
Spain	Crohn's disease and ulcerative colitis patients treated with Infliximab (IFX) or adalimumab (ADM) (anti-TNF Abs) (101)	VNTR1: 1.0	There was no association between IFX or ADM concentrations and genotype	(112)
		VNTR2: 9.9		
		VNTR3: 81.7		
		VNTR4: 0.5		

Belgium	Inflammatory Bowel Disease (IBD) patients treated with Infliximab (395) or adalimumab (139) (anti-TNF Abs)	VNTR1: 0.0	Patients with the VNTR2/3 genotype had lower concentrations of anti-TNF Abs than VNTR3/3	(124)
		VNTR2: 9.4		
		VNTR3: 90.2		
		VNTR4: 0.4		
		VNTR5: 0.1		
Holland	Guillain-Barré syndrome patients treated with high-dose intravenous immunoglobulin (IVIg) (257)	VNTR1: 0.2	There was no association between the genotype and IVIg pharmacokinetics, or clinical course and disease outcome	(125)
		VNTR2: 9.1		
		VNTR3: 89.5		
		VNTR4: 0.8		
		VNTR5: 0.4		
Belgium	Recurrent ovarian cancer patients treated with Farletuzumab (Anti-folate receptor α Ab) (470)	VNTR1: 0.0	Patients with VNTR2/3 genotype had lower concentrations of anti-TNF Abs than VNTR3/3. VNTR2/3 genotype was associated with lower infliximab and lower adalimumab exposure in IBD patients	(117)
		VNTR2: 9.4		
		VNTR3: 84.0		
		VNTR4: 0.4		
		VNTR5: 0.1		
China	Myasthenia gravis patients (334)	VNTR1: 0.0	Patients with the VNTR2/3 genotype had lower levels of endogenous IgG than VNTR3/3. All responders to IVIG treatment for myasthenia and few nonresponders were VNTR3/3. In IVIG-treated patients, endogenous IgG levels were lower in non-responders, especially in VNTR2/3 heterozygous individuals	(126)
		VNTR2: 1.6		
		VNTR3: 98.4		
		VNTR4: 0.0		
		VNTR5: 0.0		
China	Advanced non-small cell lung cancer patients treated with steady state plasma-concentration of pembrolizumab (Anti-programmed death 1 molecule) (38)	VNTR1: 0.0	VNTR3/VNTR3 homozygous patients had significantly higher steady state plasma concentration of pembrolizumab than VNTR2/VNTR3 heterozygous patients	(103)
		VNTR2: 3.9		
		VNTR3: 96.1		
		VNTR4: 0.0		
		VNTR5: 0.0		

IVIG, Intravenous Immunoglobulin; SCIG, subcutaneous immunoglobulin.

Regarding polymorphisms impacting gene expression, Sachs et al. (2006) described a variable number of tandem repeats (VNTRs) located in the promoter region (108). Each repeat consists of 37 bp, and there could be one to five repeats per allele, although more current studies have detected up to eight VNTRs (110, 111). The allele with three VNTRs (VNTR3) is the most frequent (>84%), followed by the allele with two VNTRs (VNTR2), which varies from 1.6% in individuals from China to 11.9% in individuals from Spain and Greece. Alleles with one or more than four VNTRs have the lowest frequencies (**Table 1**). Besides, Sachs and collaborators (2006) demonstrated that there is significantly less FcRn transcript, as well as decreased IgG binding capacity, in monocytes from VNTR2/3 heterozygous than in monocytes from VNTR3/3 homozygous individuals, which could indicate a dose-dependent effect in transcript quantity or protein level, rather than the activity of the FcRn, although this must be corroborated. The VNTRs have been analyzed in different populations to determine their association with some characteristics, mostly pathologies and biological phenomena such as IgG transfer. Some studies have determined that patients with the VNTR2/3 genotype had lower concentrations of therapeutic monoclonal antibodies than those who were VNTR3/3 homozygous during pregnancy (97, 109, 111–120).

In other mammals, the analysis of isolated *Fcrn* sequences from eight strains of mice showed that the distal membrane domain has at least three amino acid variants (20). In the porcine FcRn gene, exon 6 has C8526T, demonstrated by DNA sequencing analysis, which is associated with higher serum antibodies concentration against the classical swine fever virus (anti-CSFV) in three pig races, Large White, Landrace, and Songliao Black (121). Studies performed in Cynomolgus and Rhesus macaque genes showed variants S26N in the α1 domain, as well as G255S, A313S, and S355L in the α3-domain, without being relevant to the FcRn function (122). Sequencing results showed that the Rhesus macaque *FCGRT* promoter region presents, similarly to the human gene, VNTRs of 37 bp in length, ranging from 1 to 5 repeats, with variable frequencies, being VNTR4 (52%) the most frequent, followed by VNTR1 (26%), VNTR2 (13.2%) and VNTR3 (8.8%). Furthermore, several synonymous SNPs were found in exons 2-6, with no apparent relevance in function or expression (123).

In sheep, specifically in Chinese Merin, Suffolk, Romney Hills, and BELTEX breeds, three allele patterns have been reported in a 680 bp gene fragment, one of them, the least frequent, associated with the highest colostrum IgG concentration (105 mg/mL). In contrast, the most common allele had the lowest IgG average (85 mg/mL) (124). In a 1305 bp *FCGRT* fragment of genomic DNA from various cattle breeds and founders from a reference population, two SNPs in exon 3 were identified: G/A and C/A in positions 245 and 281, respectively, of the AY092412 GenBank reported sequence. In addition, three SNPs in introns 4 and 5: C/A, G/A, and C/T in the positions 314, 522, and 803 in the AY092413 GenBank sequence. These SNPs were grouped into five haplotypes, starting with intron SNPs; bovine mothers with haplotype 3 (CGCAC) had a significantly increased risk of passive transfer failure to their calves. Haplotype 2 (AGTGC) represents a lower probability of having elevated levels of passively acquired immunoglobulin through colostrum (125).

On the other hand, in Holstein-Frisian cows, four SNPs have been identified: G/A in exon 4, C/T in exon 5, C/A in intron 5, and C/T in intron 6, which were classified into five haplotypes. Haplotype 5 (ACCC) was significantly associated with a high level of IgG in colostrum (9.90 times more likely to have high colostral IgG levels). Haplotype 2 (GCAT) exhibited 2.89 times of IgG (126). These data indicated that some polymorphisms and haplotypes influence the FcRn expression and IgG levels in biological fluids.

As shown in **Supplementary Table 1**, the α-chain gene shows variability in length between species of mice, pigs, and dogs, which could be involved in different levels of expression or even function, although this should be studied.

## 3 FcRn protein

### 3.1 Protein sequence and 3D structure

The IgG Fc receptor was first isolated from the brush border of enterocyte microvillous membranes of neonatal rats (127). Two groups, using different methodologies, determined that the FcRn consisted of two polypeptide chains (**Figure 1**) of 45–51 kDa (p51) and 12–15 kDa (p14), with the smallest component being β2-microglobulin (β2m) (127, 128). The small molecule is about 11,800 Daltons and is present in almost all nucleated cells except erythrocytes. It consists of two β sheets formed by seven β chains linked by a disulfide bridge that builds a typical immunoglobulin domain. The β2m protein does not have a transmembrane region. It is non-covalently linked to the α-chain to form MHC-I molecules or similar structures, including FcRn, proteins of the cluster of differentiation group 1 (CD1), human hemochromatosis protein (HFE), and non-classical MHC class I molecule, Qa in mice, and HLA in humans (129).

**Figure 6:**
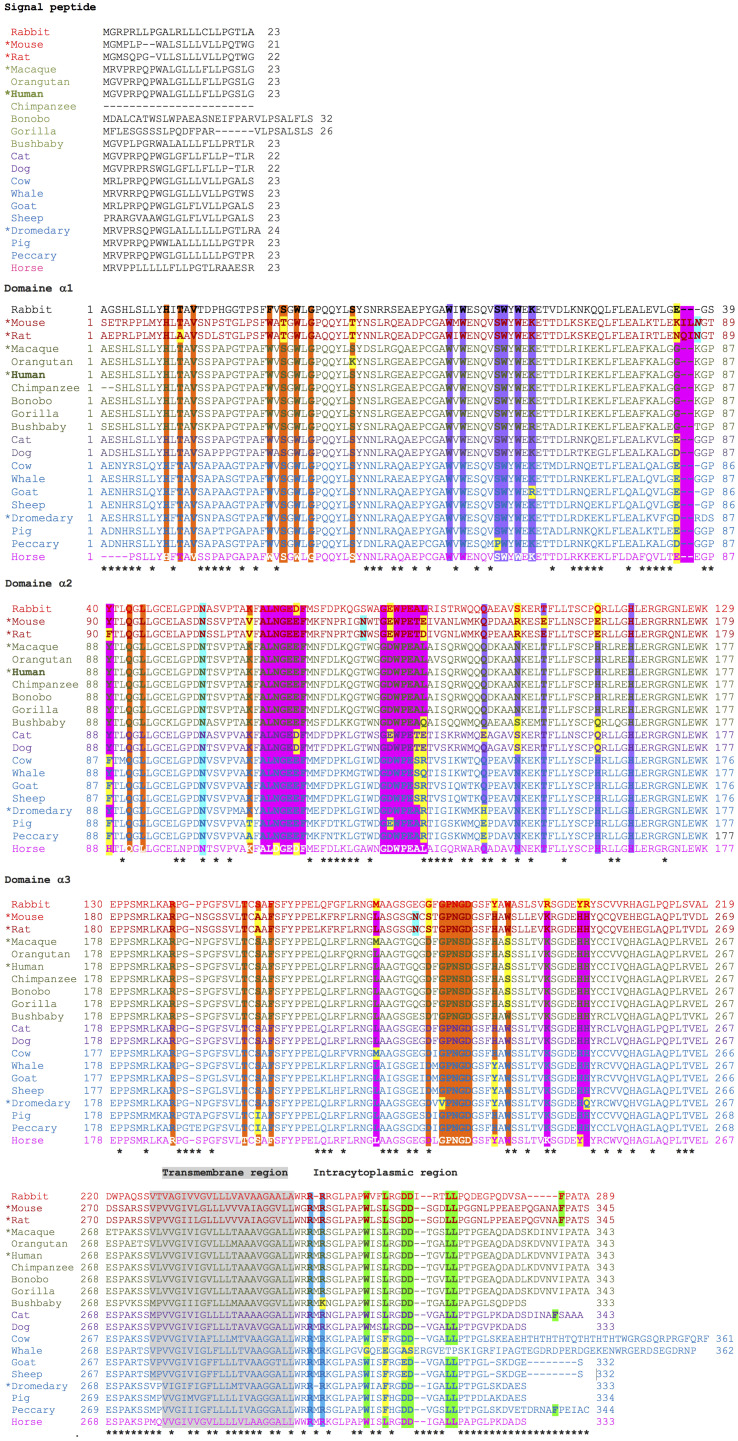
Alignment of the protein sequences and their relevant amino acids of the FcRn α-chain of twenty mammalian species. β2-microglobulin-binding sites are highlighted in orange, albumin-binding sites in violet, and IgG Fc binding sites in fuchsia. Calmodulin-binding motifs are shown in blue, and those involved in endocytosis in green. Glycosylation sites are highlighted in aquamarine. Asterisks indicate non-conserved positions.

The largest subunit was cloned in 1989 by Simister and Mostov, who showed that this chain binds to the IgG Fc portion and shares substantial similarity to the major histocompatibility complex I (MHC-I) alpha-chain (α-chain), both at the genetic and protein levels. For this reason, this proteic sequence was also referred to as the α-chain, as it comprises three extracellular domains (α1, α2, and α3), one transmembrane portion, and one intracellular region (**Figure 1**). This is highly conserved across various mammalian species (as outlined above). In humans, the main differences with class I MHC structure are that the FcRn α-chain has a narrower groove, which cannot bind peptides, and has a unique cytoplasmic domain (130). The three domains of the α-chain are immunoglobulin-like domains, corresponding to globular structures, described as flattened cylinders (β-barrels), consisting of two layers of tightly packed anti-parallel β-sheets that possess hydrophobic side chains. In humans, the α1 domain comprises amino acids 24 to 110, the α2 domain 111 to 200, and the α3 domain 201 to 299; these three α domains form the extracellular region of 273 amino acids (**Supplementary Table 1**). The three α-domains contain the eleven sites that bind to IgG, while the linkage to albumin takes place only in the α1 and α2 domains (six residues), and the union to the β2m is found in the three domains (nine amino acids) (**Figure 6**, **Table 2**). The transmembrane region consists of 24 amino acids, the intracytoplasmic region of 44, and one connecting peptide of 7 amino acids joins the α3 domain with the transmembrane region. The cytoplasmic region contains two motifs that have been associated with endocytosis due to sequence similarity to other receptors involved in this process (**Table 2**), WXXF and DXXXLL, the latter corresponds to an acid dileucine motif, which in humans correspond to Trp-311, Leu-322, Leu-323, respectively, and have been related to the interaction with endocytic vesicles (26). An exchange of tryptophan for alanine reduced the endocytosis of the FcRn (131). Data from FcRn mutant mice revealed that the Leu-314 residue is essential for tryptophan-based endocytosis signaling, while Asp-317 and Asp-318 are essential for dileucine signaling. In other systems, these motifs bind to the specific m-subunit of the AP-2 clathrin adapter complex, whose interactions allow the assembly of clathrin-coated vesicles and endocytosis progression (131, 132). In the central binding site of the FcRn α-chain, three highly conserved amino acids, Ile-253, His-310, and His-435, appear crucial in IgG serum half-life in Balb/c mice. Although mice are not the ideal model to predict the half-life of IgG in humans, since they present essential differences in the binding to murine and human FcRn, it has allowed the establishment of the role of some relevant amino acids in the protein structure (133).

**Table 2:** Key amino acids of the human FcRn **α**-chain in binding to **β**2m, albumin, and IgG Fc.

Binding molecule to FcRn α-chain	FcRn α-chain domain	Amino acid exchange site		Amino acid clasification		Species
**β2m**	α1	Thr12 →	Ala12	UP→	ANP	Mo
		Ser27→	Thr27	UP→	UP	Mo, Rt
		Ser37→	Thr37	UP→	UP	Mo, Rt
			Lys37		PP	Or
	α2	Lys109→	Val111	PP→	ANP	Mo, Rt
			Thr109		UP	Pi
			Ala109		ANP	Pc
	α3	Ser200→	Ala202	UP→	ANP	Mo, Rt
			Ile201		ANP	Pi, Pc
		Asp225→	Ser227	NP→	UP	Mo, Rt
			Gly227		ANP	Rb
		Gly227 →	Val228	ANP→	ANP	Dr
		His235→	Tyr237	PP→	RNP	Rb, Wh, Go, Sh, Dr, Ho
		Trp237 →	Ser237	RNP→	UP	All primates
**Albumin**	α1	Ser58→	Pro58	UP→	ANP	Pc
		Lys63→	Arg63	PP→	PP	Go
	α2	Gln144→	Glu144	UP→	NP	Ca, Pi, Pc
			His144		PP	Dr
		Asn149→	Ser149	UP→	UP	Rb, Bs, Ca, Do
			Arg151		PP	Mo, Rt
		Thr153→	Glu155	UP→	NP	Mo
		His161→	Gln161	PP→	UP	Rb, Bs, Ca, Do
			Glu163		NP	Mo
**IgG Fc**	α1	Gly84→	Glu84	ANP→	NP	Rb, Ca, Cw, Wh, Go, Sh, Pi, Ho
			Lys84		PP	Mo
			Asn84		UP	Rt
			Asp84		NP	Do, Dr, Pc
	α2	Tyr88→	Phe90	RNP→	RNP	Rt
			Phe87		RNP	Cw, Go, Sh, Pi, Pc
			His88		PP	Ho
		Asn113→	Asp113	UP→	NP	Ho
		Glu116→	Asp116	NP→	NP	Ca, Do, Ho
			Asp118		NP	Rb
		Asp130→	Glu130	NP→	NP	Ca, Pi
			Glu132		NP	Rb, Rt, Mo
		Ala134→	Thr136	ANP→	UP	Mo, Rt
			Thr134		UP	Ca, Do
			Ser133		UP	Cw, Go, Sh
			Ser134		UP	Wh
		Leu135→	Glu137	ANP→	NP	Mo
			Glu135		NP	Ca, Do
			Asp137		NP	Rt
			Gln137		UP	Bs, Wh
			Arg134		PP	Co, Go, Sh
			Arg137		PP	Dr, Pi, Pc
	α3	Leu218→	Met219	ANP→	ANP	Rb, Ma, Cw
		Lys243→	Arg245	PP→	PP	Rb
		His248→	Tyr250	PP→	RNP	Rb, Ho
		His249→	Arg251	PP→	PP	Rb
			Arg249		PP	Dr

UP, Uncharged polar; PP, Positive polar; NP, Negative polar; ANP, Aliphatic nonpolar; RNP, Aromatic nonpolar.

Mo, mouse; Rt, rat; Or, orangutan; Pi, pig; Pc, peccary; Rb, rabbit; Dr, dromedary; Wh, whale; Go, goat; Sh, sheep; Ho, horse; Ca, cat; Bs, bushbaby; Do, dog; Cw, cow; Ma, macaque. Each section of the Table is highlighted with the color corresponding to each of the molecules with which the FcRn alpha chain, β2m (in orange), the IgG Fc region (in fuchsia), and albumin (in violet) interact, as can also be seen in **Figure 6**.

Furthermore, using FcRn mutants and CD8/FcRn chimeric molecules, it has been identified that the insertion of the highly conserved sequence, GLPAPWISL, in the cytoplasmic tail of the α-chain is directly related to efficient endocytosis and trafficking to recycling endosomes, and at low levels to late endosomes. The change with alanine residues (AAAAAWISL) produced endocytosis of the CD8/FcRn chimeras towards early endosomes, with predominant transfer to late endosomes, not to recycling endosomes (134). These amino acids would be related to the recycling and transport of IgG and albumin, which appear to depend on the cell type and the specific gene regulation mechanisms, but this needs to be studied.

### 3.2 FcRn binding to IgG and albumin

**Figure 7:**
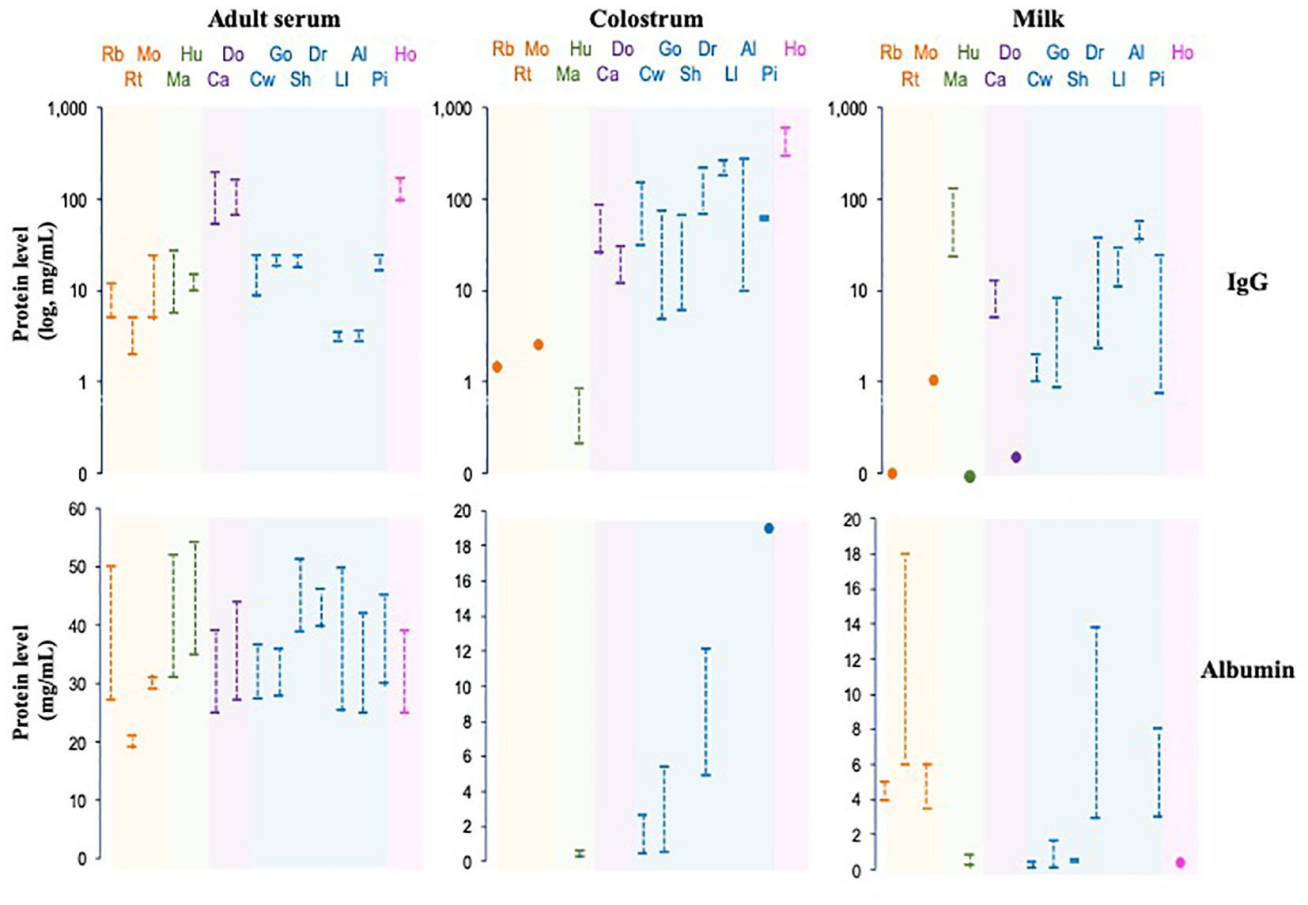
IgG and albumin levels in serum, colostrum and milk of some mammalian species. The species are grouped phylogenetically by colors: Rb, rabbit; Rt, rat; Mo, mouse; Ma, macaque; Hu, human; Ca, cat, Do, dog; Cw, cow; Go, goat, Sh, sheep; Dr, dromedary, Ll, llama; Al, alpaca; Pi, pig; Ho, horse.

Immunoglobulin G (IgG), one of the most abundant proteins in serum, accounting for 10–20% of total proteins and whose levels vary between species (**Figure 7**), is a glycoprotein composed of 82–96% protein structure and 4–18% carbohydrates. IgG is the main serum antibody of the five classes of immunoglobulins in humans, with multiple functions as a potent and versatile mediator of host protection. This immunoglobulin generates its biological effects through the interaction of the Fc fraction with complement proteins and specific cellular receptors, such as FcγR (135, 136).

Albumin is the most abundant protein (~50%) in the mammalian serum; however, the concentration varies widely among species, as in different biological fluids, like colostrum and milk (**Figure 7**). Some essential properties of albumin include transport, ligand binding, distribution, and metabolism of various compounds since it has esterase-like enzymatic activity, which allows the conversion of some prodrugs into active therapeutic agents. Albumin is a negatively charged, non-glycosylated protein consisting of 67% α-helices connected through flexible loops that form three homologous domains (DI, DII, and DIII). Each domain contains hydrophobic pockets that bind and transport small, insoluble molecules, such as fatty acids, hormones, toxins, and certain drugs, throughout the body. In humans, albumin has a serum half-life of three weeks, like total IgG (137, 138).

A large body of published data demonstrates that FcRn binds to IgG and albumin at distinct, nonoverlapping sites (**Figure 1**). Neither molecules compete nor cooperates with the other in binding. The first studies in humans and in rodents, like mice and rats, about the FcRn structure showed a 2:1 FcRn: Fc complex, i.e., two molecules of FcRn binding to one IgG molecule, which is more common than the 1:1 complex (139–141). The co-crystals of FcRn and Fc demonstrated two distinct orientations of the complex, which differ by almost 90 degrees. The first, called the “lying down complex,” involves an asymmetrical contact between both molecules, which implies that the Fc is primarily in contact with one molecule of the FcRn; this binding could function as an endocytosis signal, followed by the recruitment of the second FcRn molecule to assemble the trimer. The second model, the “standing up complex,” is formed by a symmetrical binding to Fc without contact between the two receptors. Its complex is oriented with its long axes perpendicular to the plasma membrane; this is the most accepted model (**Figure 1**) (142–145). The FcRn could bind simultaneously on opposite sides to albumin and IgG, although the stoichiometry is different; IgG can interact with two receptors simultaneously, while albumin binds to only one (146, 147). Further, the α and the β2m chains have non-covalent molecular interactions with each other and both ligands; the specific amino acids involved in each interaction are described in **Table 2**. As mentioned, when IgG interacts with FcRn, if the complex could display a “lying down” orientation, this would allow the binding of two FcRn molecules on opposite membranes in tubular vesicles to one IgG. However, the simultaneous union of albumin to FcRn, as observed on a cell-free surface plasmon, might not be compatible with the ‘lying down’ orientation of the FcRn-IgG-albumin complex on opposite membranes of tubular transport organelles due to a steric hindrance. As a result, albumin transcytosis and recycling may be hampered in the presence of IgG. However, it has been described that the vascular endothelium and the pulmonary epithelium, which express FcRn, can carry out transcytosis of both albumin and IgG effectively, although simultaneous transport by a single molecule or by a pair of FcRn molecules has not been demonstrated.

Furthermore, no evidence of FcRn-mediated albumin transportation has been found across some types of endothelia, lung epithelium, or placental tissue, except in the liver. However, some data in *in vitro* models have reported the opposite. Thus, these mechanisms have yet to be elucidated (148, 149). It is well known that FcRn binds to IgG Fc with high affinity, mainly at slightly acidic pH (6.0–6.5), and exhibits no detectable union to this ligand at neutral or basic pH, except for IgG2b antibodies in mice and some allotypes of human IgG3, which show weak binding at neutral pH. According to Vaughn and Bjorkman, FcRn is more thermally stable, and its light chain dissociation rate is an order of magnitude slower at pH 6.0 than at pH 8.0. The affinity of FcRn by Fc of IgG had been determined and is higher at pH 6.0 than at pH 7.0 (K_D_~10 nM vs K_D_~8 mM) (142, 150). In addition, the two FcRn molecules bind to IgG homodimer with equal affinities at the independent sites (151). The affinity of the FcRn-IgG interaction could also vary depending on the species and IgG subclasses (see below). The albumin interaction with human FcRn showed moderately high affinity at pH=5.0 (K_D_=0.2 - 0.7 mM) but was negligible at pH=7.0 (K_D_=34–408 mM) (148). As described above, the affinities of the interaction between FcRn and IgG and albumin are crucial for the half-life of both molecules in the body, as the ability of FcRn to recycle them contributes to prolonging their half-life.

#### 3.2.1 FcRn affinity to IgG subclasses

In humans, which is the most studied species on this topic, four IgG subclasses, IgG1, IgG2, IgG3, and IgG4 were identified, based on structural, antigenic, and functional differences in the constant region of their heavy chain, specifically in the CH1 and CH3 domains (135, 152). Human IgG subclasses were numbered according to the order of their serum levels of healthy Western European individuals (IgG1>IgG2>IgG3>IgG4). IgG1 comprises 60-65% of total IgG and is predominantly responsible for the thymus-mediated immune response against polypeptide proteins and antigens. IgG1 can interact with Fc receptors on phagocytic cells and with C1 complex, activating the complement cascade. IgG1-dependent immune response is frequently generated simultaneously with IgG3 and sometimes with IgG4. IgG2 covers 20-25% of the IgG and is the predominant antibody response against carbohydrate/polysaccharide antigens. IgG3 encompasses around 5-10% of total IgG, although there are variations among human populations, and it plays a vital role in immune responses against protein or polypeptide antigens. IgG4 corresponds to less than 4% of total IgG and does not bind to polysaccharides; this precise role is still under discussion, as it is the only subclass that does not fix complement (136). **Supplementary Table 3** shows some data on IgG subclasses in humans and other mammals, including levels in serum, colostrum, and milk, which seem to be closely related to their binding affinity to FcRn and probably, to their specific activity in different organs or cell types, which has not been thoroughly explored, but could be the reflection of the various levels of IgG subclasses in biological fluids (145, 153–204). Half-life varies widely among IgG subclasses (**Supplementary Table 3**), probably due to the peptide structure influencing FcRn affinity, recycling, and transport. The amino acid structures of the four human IgG subclasses are very similar, with more than 90% homology and differences in the number of disulfide bonds and the length and flexibility of the hinge region. The mobility and flexibility of the antibody F(ab) and Fc portions are mainly controlled by the CH1 domain and the hinge region. Each IgG subclass has a unique profile for antigen binding, immune complex formation, complement activation, effector cell activation, half-life, placental transport, and binding to molecules and receptors FcγR, including FcRn (135, 152). The residues of the CH2 domain closest to the hinge region are responsible for the effector functions of the antibodies since there is an overlapping binding site for the C1q of complement and for the FcγRs. The highly conserved N-glycosylation site 297, located at the interface between the CH2-CH3 domains, is responsible for changes in the Fc quaternary structure, associated with a position more or less exposed to the binding site of the FcγRs and modulates specific immune responses in humans. The interface between the CH2-CH3 domains also contains the binding site for FcRn (135). In addition, IgG subclasses exhibit allelic variation. Genetic analyses identified a large number of polymorphisms, mainly in structural determinants. The allelic forms of IgG1 reported are 6, the main ones being G1m(z, a), G1m(f), and G1m(f, a). Allelic forms of IgG2 include two variants, G2m and G2m23, which, along with IgG4, nG4m(a) and nG4m(b), serve as examples of IgG variants without true allotypic determinants. For IgG3, many allelic forms are known, at least 16, with the most critical generating amino acid changes (205). However, the influence of allelic variations on FcRn binding has not been analyzed.

The affinity of FcRn for IgG subclasses is unknown in many species; in humans, it has been related to the serum levels and half-lives of each subclass: IgG1 is the one binding with the highest affinity, followed by IgG2, IgG3 (these subclasses depend on the population), and finally IgG4 (104, 206). In mice, it has been shown that the homologous FcRn binds IgG1 with high affinity (K_a_~8x106 M-1). IgG2a and IgG2b are known to bind, apparently with high affinity, while IgG3 binds FcRn with low affinity, although the values have not been reported (207, 208). Therefore, these characteristics and effects could be similar in other organs and specific cell types in function and levels of IgG subclasses.

#### 3.2.2 FcRn binding to immune complexes

Immune complexes are composed of antibody and antigen molecules that act as a single unit; they interact by non-covalent, stable, and specific interactions without any irreversible chemical alteration in either of the two molecules. Once bound, the immune complexes can act in many processes, such as the complement cascade, protease processing, receptor recognition, opsonization, phagocytosis, etc. (209). FcRn can bind IgG-antigen complexes; this phenomenon was described before the receptor was isolated and characterized. The process was determined in the enterocytes of the small intestine of lactating rats, where the immune complexes are transported from the breast milk to the blood (210). It has been seen that the immune complexes that bind to the FcRn are varied, including some small ones, such as IgG bound to ovalbumin (OVA), and even microorganisms, such as cytomegalovirus or HIV, pathogens using natural mechanisms to cross biological barriers (211, 212). The bound antigen’s physicochemical properties influence the immune complexes’ direction inside the cell since they induce conformational changes in the Ag-IgG and FcRn tertiary complex. The magnitude of these structural changes is directly related to the affinity of the antigen with IgG and, in turn, with the FcRn (213). Once internalized, these complexes can be transported, for example, through the epithelial or endothelial cells of different organs, or they can be sent for degradation in lysosomes or cross-presentation of antigens (214).

### 3.3 Protein similarities/dissimilarities among species

Based on multiple alignments and a neighbor-joining tree conducted using the amino acid sequences of the FcRn α-chain from the twenty selected mammalian species (**Figures 4**, **6**), a general sequence conservation is observed, including amino acids that interact with other polypeptide chains, such as β2m (in orange), the IgG Fc region (in fuchsia), and albumin (in violet), along with amino acids located in different regions (**Table 2**). Most species retain these essential binding amino acids, albeit with variations, such as in rats, where the changes are more significant, followed by mice and rabbits regarding β2m-binding amino acids and similar sequences for albumin binding (**Table 2**). The Fc-binding amino acids within the α1 and α2 domains exhibit more variability among species, with notable changes seen in rabbits and primates. The FcRn α-chain in humans possesses two glycosylation sites (in aquamarine), conserved across all analyzed species except mice and rats, which have four sites. This difference could potentially influence the IgG binding capacity of different species, as murine FcRn has been shown to bind to a wide range of IgG types, unlike human FcRn (215). Key motifs responsible for binding to calmodulin (in blue) and involved in endocytosis (in green, as described previously) are conserved, except for one site where members of the artiodactyl group exhibit a change (leucine to phenylalanine), which could impact recycling and transport activity, as well as IgG and albumin levels in various biological fluids (**Figure 6**; **Supplementary Table 3**). The signal peptide located in the N-terminal region, typical of most peptide chains, remains conserved in length and sequence (ranging from 21 to 23 amino acids), containing several hydrophobic amino acids for translocation. The bonobo sequence exhibits the longest signal peptide, followed by the gorilla (32 and 26 amino acids, respectively), with distinct sequences (**Figure 6**).

Regarding the central portion of the protein, total size and domain architecture are similar among species (**Figure 6**). A degree of homology exists at the gene, cDNA, and protein levels across species, ranging from 50% to over 90%. Variations have been described within strains or breeds of certain species, such as pigs or mice, in terms of RNA length and transcribed region, seemingly without significantly impacting the final length of the amino acid chain or receptor function (**Figure 6**; **Supplementary Table 1**). Despite these similarities in gene, mRNA, and protein sequences of the FcRn α-chain across species, differences are observed in various biological aspects. For instance, the transfer of IgG from mother to offspring and levels and half-life of IgG and albumin in different fluids vary among species with available data, suggesting potential differences in regulation due to hormonal, immunological, or other biological factors. Interestingly, sequence similarities among analyzed mammalian groups are primarily maintained within species of the same placentation type, which could influence the mechanism of maternal immune transfer to offspring, as described below (**Figure 4**) (20, 216). Human IgG exhibits an unusually long half-life in circulation (18 to 23 days) compared to other serum proteins. This is attributed to its interaction with FcRn, which protects IgG from lysosomal degradation by endothelial cells in direct contact with blood (135). Significant variation in IgG half-life is observed across mammalian species, ranging from one day in cats to 25 days in horses, mirroring similar trends in albumin (**Supplementary Table 3**). The half-life of albumin and IgG could vary in different biological fluids of the different species, mainly due to specific transport and recycling mechanisms. In serum, both molecules have a specific half-life, due to the FcRn-mediated recycling. In other biological fluids, albumin and IgG may have a slightly different half-life due to factors such as concentration and transport, which would be dependent on receptor expression and cellular regulatory mechanisms of the FcRn.

#### 3.3.1 Comparative analysis of FcRn in immunity transfer across species

The passive transfer of maternal immunity to offspring, which facilitates the transport of IgG across the placenta or through colostrum, is mediated by FcRn and varies significantly among species, primarily influenced by differences in placentation, gestation length, and species-specific adaptations.

In humans and nonhuman primates (e.g., macaques, chimpanzees), FcRn is the primary receptor mediating IgG transfer across the placenta. Studies in rhesus macaques have shown that FcRn-enhancing mutations in monoclonal antibodies increase their transfer to the fetus, highlighting the receptor’s critical role (217, 218). The expression of FcRn in placental and fetal tissues is a key determinant of IgG transfer efficiency across species. In humans, placental FcRn expression progressively increases during gestation, especially after the 34th week, ensuring adequate passive immunity for preterm infants (219). Interestingly, recent evidence suggests a dual receptor model in primates, with FcγRIIb possibly contributing to IgG transfer alongside FcRn, although the latter remains the primary mediator (217, 220). In the case of rabbits, FcRn-IgG transfer is preferentially prenatal via the inverted yolk sac splanchnopleure, where it facilitates prenatal IgG transport to the fetus (25, 221).

Colostrum and milk are vital compounds female mammals produce to nourish and provide passive immunity to offspring while their immune and digestive systems develop. Colostrum quality and composition differ among mammalian species. They contain various host resistance factors, such as complement, macrophages, lymphocytes, lactoferrin, lactoperoxidase, lysozymes, and antibodies, which protect against pathogens (175, 222). Unlike humans, nonhuman primates, and rabbits, animals such as rodents, cats, and dogs primarily transfer IgG to their offspring postnatally through colostrum. However, FcRn remains crucial for IgG transport within the yolk sac and placenta during gestation, reflecting a dual role for FcRn in mediating IgG transfer both pre- and postnatally (221, 223).

In contrast to species with efficient prenatal IgG transfer, many large domestic animals, such as cattle, sheep, and goats, possess placental structures that prevent significant transplacental IgG passage, making them entirely dependent on colostral IgG for neonatal immunity. In these species, FcRn is expressed in the neonatal small intestine, where it actively mediates IgG absorption from colostrum to provide essential immune protection (26, 224).

Species-specific mechanisms further distinguish IgG transfer modalities and FcRn functions. In pigs, FcRn is essential for IgG homeostasis and absorption in the neonatal intestine but does not mediate IgG secretion into colostrum, which occurs through an FcRn-independent pathway (225). Horses exhibit a similar pattern to ruminants, relying exclusively on colostral IgG absorption mediated by FcRn in the neonatal gut (226). Notably, the concentration of IgG in colostrum varies widely across species, with ruminants and pigs producing particularly IgG-rich colostrum to compensate for the absence of prenatal transfer. This variability is matched by coordinated FcRn expression in mammary glands and neonatal intestines to maximize IgG transfer efficiency (26, 224).

Finally, limited data are available on the function of FcRn in marine mammals. However, their placentation type (epitheliochorial) suggests that IgG transfer may occur via colostrum rather than the placenta (226).

As described, the transfer of passive immunity varies among mammalian species and is classified into three groups based on immunoglobulin transfer processes (**Figure 7**) (175, 222). These differences likely reflect variations in FcRn expression levels in organs involved in IgG transport, such as the placenta, yolk sac, mammary gland, and newborn intestine. Furthermore, variations in the number and characteristics of IgG subclasses are found among mammalian species, with binding affinity to FcRn closely related to levels and half-life in biological fluids (145). Variations in IgG subclass properties likely impact their efficacy in different organs, highlighting the influence of FcRn in albumin and IgG homeostasis and immunity across species.

## 4 Functions of FcRn

**Figure 8:**
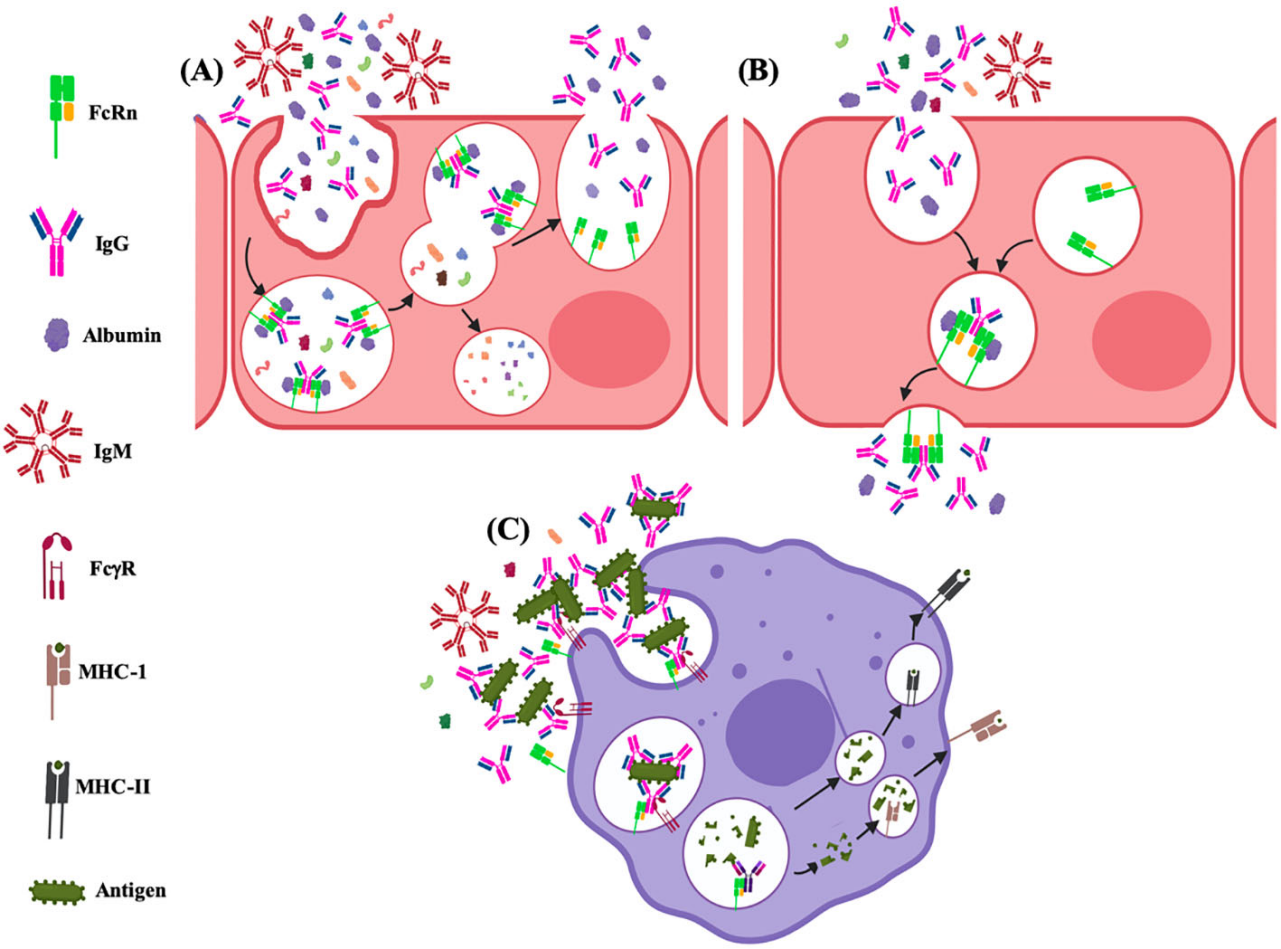
Functions of the FcRn at cell level. **(A)** recycling and **(B)** transcytosis of albumin and IgG; and, **(C)** roles on immune activation via antigen cross-presentation and immune surveillance.

**Figure 9:**
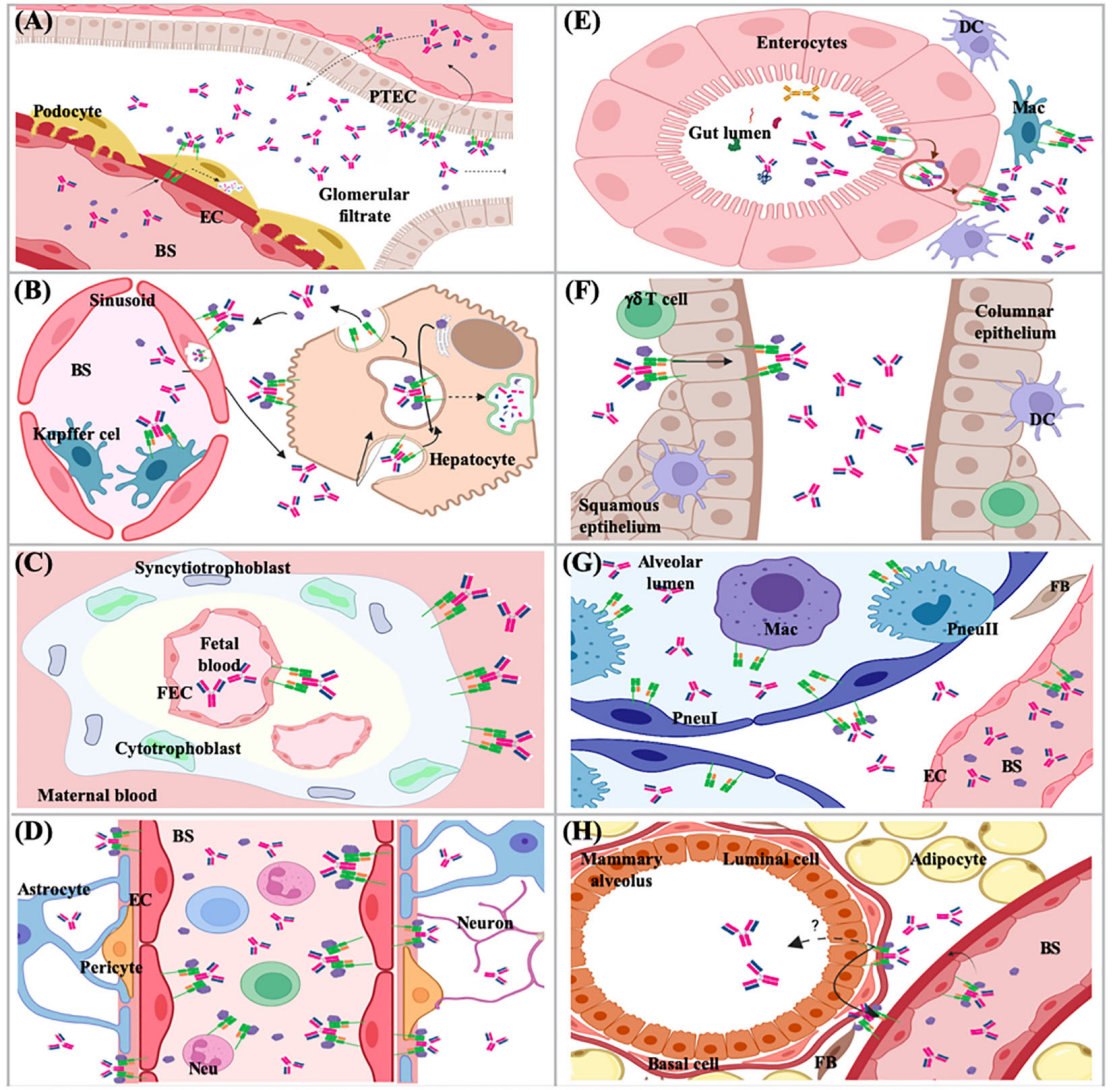
Functions of the FcRn in different tissues and organs. **(A)** kidney; **(B)** liver; **(C)** placenta; **(D)** brain; **(E)** small intestine; **(F)** female genital tract; **(G)** lung; **(H)** mammary gland. PTEC, proximal tubular epithelial cell; EC, endothelial cell; BS, bloodstream; DC, dencritic cell; Mac, macrophage; FEC, fetal endothelial cell; Pneu, pneumocyte; FB, fibroblast; Neu, neutrophil.

The FcRn has prominent and well-established roles in the homeostasis of serum proteins, transcytosis, immune response activation and regulation, phagocytosis, and antigen presentation (**Figures 8**, **9**). These functions depend on the specific cell type and tissue due to several subcellular phenomena related to its formation, intracellular transport vesicles´ fusion, and trafficking to the plasma membrane (227).

### 4.1 Serum proteins homeostasis

The IgG and albumin homeostasis processes have been described since 1950, although the specific cell type and molecular mechanisms that carried them out were unknown (228–230). The FcRn is expressed in different cell types; however, the only cells that recycle IgG are endothelial cells of blood vessels, professional antigen-presenting cells and derived from bone marrow, while albumin salvaging has been determined in endothelial cells and hepatocytes, the organs that mainly participate in the regulation of IgG and albumin levels in the organism are kidney and liver (**Figures 8**, **9**) (231–233). In the kidneys, FcRn is expressed in podocytes, the proximal tubular epithelium’s brush border, and vascular endothelial (**Figure 9**) (234, 235). In podocytes, FcRn participates in the internalization of IgG and albumin, reaching the glomerular basement membrane (GBM). This process was determined by *in vitro* and *in vivo* studies. Mice lacking FcRn accumulated IgG in the GBM as they aged; tracer studies showed a delay in eliminating antibodies from the kidneys, which generated a saturation in the clearance mechanism and nephrotoxicity (236). Therefore, podocytes play an active role in GBM protein clearance, and sustained disruption of plasma protein trafficking alters glomerular structure. More recent data indicates that the albumin and IgG trafficking pathways in podocytes are different (237, 238). In human renal proximal tubule epithelial cells (hRPTEC), FcRn participates in IgG and albumin homeostasis since it rescues them from the tubular fluid (235). According to Kobayashi et al., FcRn in RPTEC is distributed on the cell surface and in the cytoplasm. IgG is transported through these cells in a bidirectional manner. Therefore, the transport of IgG from the apical to the basolateral surface, mediated by FcRn, by reabsorption of the tubular fluid, plays a vital role in IgG homeostasis. The passage of IgG from the basolateral to the apical surface, similar to that described for intestinal epithelial cells, would affect mucosal immunity. IgG secreted into the lumen can be locally sourced and selectively transported across mucosal barriers in the same way as IgA (234, 239).

The liver is composed mainly, between 70-80%, of the specialized and polarized epithelial cells, the hepatocytes, which have an apical side that converges towards the bile canaliculi and a basolateral side in contact with the blood. Western blot of the canalicular (apical) and sinusoidal (basolateral) plasma membranes of adult rat hepatocytes demonstrated FcRn expression, enriched on the superficial part of the canalicular membranes. FcRn was functional because it bound to IgG Fc fragments at pH 6.0 but not 8.0 (240). In mouse liver sections, an intense FcRn staining was found on the surface of hepatocytes, limited to the cell membrane adjacent to the hepatic sinusoids and in sinusoidal lining cells, including endothelial and Kupffer cells. The endothelium lining the central vein and portal vasculature was negative for FcRn staining (231). The liver receives more blood than the kidneys six times daily due to the high supply of arterial blood from the heart and venous blood from the digestive tract. Thus, large amounts of IgG and albumin are in direct contact with hepatocytes, which have been determined to be endocytic ally active. It is surprising that even though the amount of IgG in the blood is five times greater than that of IgA, less IgG reaches the bile than secretory IgA. Hepatocytes produce albumin; however, the albumin level in the blood is 100 times higher than that found in bile (241). Some studies indicate that FcRn in hepatocytes could be a receptor that protects IgG from catabolism in these endocytic cells, the protection of IgG results from recycling the receptor towards the same surface of the apical membrane (149). As mentioned, albumin synthesis takes place in hepatocytes. *In vitro* models with polarized cells have shown that FcRn regulates basal recycling and bidirectional transcytosis of albumin, mainly directing the physiological release of newly synthesized albumin towards the basal medium, preventing it from going to bile. These properties make the liver FcRn mediate the release and maintenance of albumin in the circulation and, therefore, its biodistribution (149, 242, 243). Likewise, it has been shown that the absence of FcRn in the liver leads to hypoalbuminemia since it causes albumin retention within hepatocytes and increases the excretion of bile albumin (243).

To the best of our knowledge, the interaction of FcRn with other cellular receptors is unknown, it has only been seen that it participates in the cross-presentation of antigens, which leads to a specific immune response (see the section 4.4), consequently, further studies are required.

### 4.2 Transcytosis

In certain cell types of various organs, the FcRn carries out IgG transport (**Figures 8**, **9**), which can be unidirectional, as in the syncytiotrophoblast of the human placenta (244–246), in the specialized endothelium of the eye or brain (247–249) or bidirectional as in the epithelium of the kidney, intestine and female genital tract (**Figures 8**, **9**) (250–253). The FcRn could transport both IgG alone and form immune complexes. The transport of monomeric IgG in various organs allows the antibodies to participate in immunity transfer. Thus, the antibodies can detect the antigens or microorganisms and generate a specific response (immune surveillance). In contrast, in the case of the transport of immune complexes, it favors their direct detection by cells of the specific immune response (activation) and their subsequent regulation or the direct presentation (see later) (254). In mice, it has been observed that the lack of the *Fcgrt* gene leads to the inability to recycle IgG or albumin and degrade IgG at an increased rate, resulting in low levels of both molecules in plasma. Furthermore, these animals cannot transport IgG across epithelial barriers, such as the intestine and the placenta. Likewise, *B2M* gene deficiency also affects the half-life of serum IgG (247). However, the subcellular mechanisms that direct this transport in different cell types are still unknown.

### 4.3 Immune response activation and regulation

The FcRn plays a crucial role in activating and regulating the immune response, and its function begins with the transport of immune complexes. This process was initially identified in the epithelial cells of the small intestine of lactating rats. IgG immune complexes in breast milk were transported into the bloodstream without sufficient data that this could immunize or induce future antigen tolerance in neonatal rats (250, 255). It is currently well known that FcRn can transport immune complexes across the epithelial barriers of different organs and deliver them to antigen-presenting cells, such as dendritic cells. Then, the antigen is processed in APCs, generating peptides loaded onto MHC-I and MHC-II. This process allows the synchronous activation of CD4^+^ and CD8^+^ T cell responses against antigen, primarily in IgG-rich tissue compartments. This leads to homeostatic immune activation and induction of an inflammatory response upon antigen exposure (254, 256). Studies in mice have shown that the expression of FcRn in cells of bone marrow origin contributes to the protection of both monomeric IgG and circulating immune complexes. In addition to regulating the levels of these immune complexes, the FcRn expressed in APCs also promotes their intracellular targeting to specific compartments for their processing and antigen presentation through the MHC class II to CD4^+^ T cells or cross-presentation through the MHC class I to CD8^+^ T cells, in addition to the secretion of inflammatory cytokines. These data suggest that FcRn participates in the inflammatory processes by preventing IgG and immune complexes’ degradation, allowing immune complexes to mediate innate and adaptive immune functions (257). Moreover, transgenic mice that overexpress FcRn and immunized with OVA, synthetic peptide based on the conserved hemagglutinin subunit 2 (HA2) or vaccinated against influenza presented serum antigen-specific IgG increasing, with unexpected augment of IgM, larger spleens, and increased numbers of antigen-specific plasma and B cells, granulocytes and dendritic cells. Neutrophils from these transgenic mice phagocytized IgG immune complexes more efficiently than control mice, with a strong expression of FcRn in peritoneal macrophages and bone marrow-derived dendritic cells and better antigen presentation by dendritic cells (258–260). Taken together, these results demonstrate that FcRn is capable of transporting antigens, or even bacteria and viruses, in the form of immune complexes, which are presented, generating a specific immune response in regional lymphoid structures, involving defense and immunoregulatory functions on mucosal surfaces and conferring protection against certain microorganisms (253, 261, 262).

Some controversial data have indicated that human FcRn could facilitate the transepithelial transport of IgE immune complexes. The authors suggest that maternal IgE could cross the placenta or the intestine of newborns, mediated by FcRn as IgG anti-IgE/IgE immune complexes. They also indicate that this process is related to beneficial or pathogenic processes in babies and could serve all antibodies. However, this needs experimental corroboration (263, 264). Likewise, the pathogenic effects of transporting specific immune complexes in some individuals with autoimmune diseases can be ameliorated using FcRn blockers (265, 266).

The immune surveillance mechanism has been described in different organs and tissues (**Figure 9**), such as the female genital tract, kidney, brain, the lung, and probably the mammary gland. In this mechanism, the epithelium’s FcRn binds to monomeric IgG. It is transposed from the apical to the basolateral side to identify antigens and transport them to trigger specific mechanisms to protect the organism (253, 256, 257, 261).

### 4.4 Phagocytosis and antigen presentation

The FcRn could favor sorting IgG into recycling endosomes to prevent degradation and avoid its direction towards lysosomes (214). Oppositely, the FcRn recognizes complexes of IgG bound to antigens and transports them to the lysosomes in which cross-presentation of antigens occurs (**Figure 8**). One of the first studies identified the binding of FcRn to IgG-OVA complexes, with the ability to transport them bidirectionally through polarized MCDK cells. OVA recognized by IgG and transported across the cells was released, retaining its antigenicity for CD4^+^ T lymphocytes. These experiments were corroborated in the epithelium of the mice’s small intestine, where immune complexes were transported into the lamina propria, processed by antigen-presenting cells, like dendritic cells (CD11c^+^), and presented to CD4^+^ T lymphocytes in local lymphoid structures (256). This is supported by *in vivo* and *in vitro* proliferation assays, which showed that an OVA epitope was more efficiently presented to T cells in wild-type mouse dendritic cells than FcRn-deficient dendritic cells (267). In murine dendritic cells (CD8^−^CD11b^+^), cross-linking of multivalent immune, not monomeric, complexes by FcRn was identified, being more intense at low doses of antigen, which triggers specific mechanisms that result in immune complexes trafficking towards lysosomal compartments, where the antigen it is processed into peptide fragments, loading into molecules of the MHC-I or -II, to present them to CD4^+^ or CD8^+^ T cells (268). This FcRn-mediated antigen cross-presentation process has significant consequences for homeostatic immune activation and the induction of inflammatory responses following antigen exposure (254). In human microvascular endothelial cells (HMEC-1), the process by which FcRn can transport immune complexes to lysosomes for antigen processing was studied. The receptor alone or linked to monomeric IgG is usually positioned in recycling tubules emerging from early endosomes. FcRn bound to immune complexes was not visualized on microtubules directed toward the receptor, which was not bound to its ligand. These results are similar to those found for the transferrin receptor, so a general mechanism of action was suggested. When the cytoplasmic part of the FcRn was removed or replaced, the shunting of the complexes toward lysosomes after cross-linking was not prevented. The size of immune complexes appears to be one of the most important physical properties for classifying them into recycling or targeting pathways to lysosomes. However, the specific mechanisms have not been elucidated (214).

Despite its regulatory and transport functions for IgG and albumin, evidence suggests that FcRn may promote pathogenic events, as it cannot distinguish between beneficial and harmful IgG or immune complexes. In this context, several studies have established the role of FcRn in IgG-mediated autoimmune diseases, as it can keep pathogenic antibodies in circulation by recycling or passing pathogenic IgG through cell layers, allowing them to recognize their specific antigens. Among the diseases associated with FcRn, myasthenia gravis, systemic lupus erythematosus, pemphigus vulgaris, inflammatory bowel disease, and immune thrombocytopenia have been documented (269–272). Another alteration associated with FcRn’s function is the persistence of pathogenic immune complexes. For instance, it has been described as being involved in the formation and deposition of immune complexes in the kidney sub epithelium, which causes lesions, or in immune complex-induced tissue factor-dependent FXa activity, potentially leading to thrombosis in autoimmune hemolytic anemia and antiphospholipid syndrome (265, 266, 273, 274). These findings have been reported through studies in humans or mice; however, associations have not been analyzed in other species. Nonetheless, based on its homologous functions, it is suggested that FcRn could play a paradoxical role across different species, triggering autoimmune responses. For these reasons, in humans, and potentially affecting other species, FcRn has been proposed as a therapeutic target for treating these diseases. This proposal mainly centers on increasing the competitive pressure at the saturable FcRn binding site to reduce systemic levels or transport of IgG. FcRn-IgG binding site blockers have proven effective in inhibiting this interaction, leading to decreased circulating IgG levels (275, 276).

## 5 FcRn phylogeny

For several years it has been suggested that the similarity between the amino acid sequence and the three-dimensional structure of the protein α-chain of MHC-class I, -class II, CD1, other Major Histocompatibility Complex Class I-Related molecules (Qa-1 [HLA-E, human functional counterpart], Histocompatibility 2, M region locus 3 [H2-M3], MR1 and, MILL [MHC class I-like located near the leukocyte receptor complex]), HFE (Human Hemochromatosis Protein) and FcRn is due to they come from a common ancestor, which would be related to a general conserved function (216). The locus of MHC has been found in all vertebrates with jaws, except teleost fish. It has been proposed that the precursor of this family of proteins could have been generated by the fusion of a heat shock protein-70 (Hsp-70) peptide-binding motif and a domain of the immunoglobulin superfamily (277, 278). Likewise, it has been hypothesized that the evolution of MHC genes (MIC genes) and MHC-like genes (MIC-like genes) could be due to tandem duplication events. Developing these paralogous genes would include changes in both chromosome position and function. The diversification of the functions of this family would be due to the selective reduction of the binding groove of a functional peptide until no binding (20). All three extracellular domains, members of the Ig superfamily, of MHC-I and class I-like molecules interact with β2m (the α2 domain of MHC-II plays the role of β2m), which would have limited the evolution of this interaction surface. β2M stabilizes MHC-I and similar molecules and, facilitates the binding of antigenic peptides, maintaining their conformation for recognition by T lymphocytes (129). Remarkably, some residues at the β2m contact points are equal between the FcRn, CD1, and MHC-I molecules, i.e., aspartic acid-53. The FcRn α-chain gene shares with the CD1 genes an intron/exon structure that links the cytoplasmic region with the 3’-untranslated sequence (3’-UTR). In contrast, the cytoplasmic tails of class-I molecules are assembled from the translation of three exons. It has been found that FcRn proline-162 favors the closure of the peptide-binding groove formed between the α-helices of the α1 and α2 domains in MHC-I molecules. All CD1 molecules also have a proline at this position, whereas MHCs have a valine, leucine, or isoleucine. The general similarity, especially in α1 and α3, suggests that the FcRn shares an ancestor with MHC rather than CD1 (20, 22). The largest differences between CD1 and FcRn were found in α1, with identity around 16%. The comparison of only the α3 domain sequences shows that the *Fcrn* gene does not form a group with any other class I gene, so there is controversy, and with these data, it has been indicated that the *Fcrn* gene is not closely related to the other class I genes, including MICA, CD1 and AZGP1 (gene that codes for the Zn-α2 glycoprotein) (20). As mentioned, the α-chain sequence and domain organization of FcRn is similar to the α-chain of MHC class I, sharing 27% of amino acids in the α1 domain of mouse FcRn, 23% in α2 domain and 34% in α3 (279). Likewise, it has been suggested that the FcRn diverged from the MHC from a more recent common ancestor of lizards and mammals (129).

Two phylogenetic trees were constructed for cDNA and protein chains to determine homology and represent an approximation of the evolutionary relationship between the complete sequences of the twenty species analyzed. The phylogenetic trees were constructed using the neighbor-joining method in the EMBL-EBI Clustal Omega program (**Figures 3**, **4**). Clustal Omega calculates sequence homology using a progressive multiple alignment approach, starting with pairwise alignments and calculating the distance between all pairs. It then generates a tree based on genetic distances to construct a global alignment, and finally, a tree is built with the final multiple alignments. Furthermore, Clustal Omega minimizes the total distance between sequences in the final alignment, resulting in a more accurate alignment reflecting the evolutionary relationship between the sequences (280, 281). As seen in **Figures 3** and **4**, there is an organization of both cDNA and protein sequences of the different species according to their taxonomic order and type of placenta, indicating that, although the FcRn is a common and conserved receptor among mammals and has similar functions, it could be hypothesized that the evolution of this receptor is associated with the development of the placenta and, therefore, with the different forms of passive immunity transfer. Consequently, it can be considered that the placenta is one of the tissues where the role of FcRn is vital; however, the subtle differences in its nucleotide sequence that influence changes in the amino acids of the protein have not yet been studied in detail, and it is unknown whether they relate to the evolution that the receptor has undergone to fulfill the particularities of its function, such as the greater or lesser specificity for each of the IgG subclasses, its permanence in the bloodstream, and albumin, or, when appropriate, with the passage of IgG during lactation in the intestines of some ruminants, or with its considerable variability in permanence across different tissues. Both trees start with three branches, but they show different groupings of clusters. The cDNA tree provides a general view of the ancestry of the human receptor concerning 19 other species. Interestingly, in addition to showcasing the similarity between cDNA sequences, this figure reveals another grouping based on the type of placentation, and thus the transfer of maternal antibodies to their offspring during or after pregnancy. The main branch in the cDNA tree encompasses most of the species analyzed here, with the group closest to humans being the primates, as expected. Additionally, there is a closer proximity of the human sequence to that of rodents than to other mammalian species. In the second main branch of the tree, pigs and peccaries are grouped, while the most distant cluster consists of the Perissodactyls (**Figure 3**). The grouping of the different orders of mammals allows us to infer that there are discrete changes in the sequences, and these changes could be related to the evolution of each species rather than to the biological function of the FcRn. When comparing the protein sequences (**Figure 4**), it is observed that the branches shift; however, the general organization remains intact, and groups of mammals such as rodents, primates, and artiodactyls stay together. The most distant sequences belong to the rodents and the rabbit, for which several characteristics remain unknown. This analysis of protein sequences reinforces the idea that the FcRn and its biological functions are similar across all species. Changes in expression by cell type and level of expression could be regulated by various internal and external factors affecting the cells that express the receptor, aspects that should be investigated.

## 6 FcRn ontogeny

The FcRn has been detected at the mRNA and protein levels in different stages of life, from the yolk sac to the adult and in all the mammalian species. However, the levels had been reported to be different, which could be explained by the analysis technique or the level of intrinsic expression. Very few studies have analyzed the expression of FcRn during various stages of life. In the fetal stage, its presence has been demonstrated in the placenta of all the species analyzed, with differences in distribution. In humans and Cynomolgus monkeys, FcRn was identified in fetal vessels’ syncytiotrophoblast and endothelial cells, where maternal IgG transport to the fetus occurs (221, 279). In humans, the IgG transfer is inefficient in the early stages of pregnancy, begins around week 16, and increases drastically by week 22, reaching a concentration in the fetus similar to the mother by week 26. Most IgG is acquired during the last four weeks of pregnancy (279, 282, 283). In rats, FcRn has been detected in the epithelial cells of the yolk sac endoderm and the endothelial cells of the fetal vessels of the labyrinthine zone (221, 222, 284). In the murine placenta, FcRn is found only in the epithelial cells of the yolk sac endoderm (221, 222, 285). In the fetal tissues, the FcRn has been detected in endothelial and fetal cells of the cardiovascular, endocrine, and digestive systems, with the hypothesis that it also recycles IgG to maintain serum levels, similar to adults. Once individuals are born, the receptor has been identified in almost all the tissues and organs in which it has been reported for adults. However, it is still being determined if the levels are similar and if they perform the same functions. There are cases where the expression is modified at different stages of life; for example, in the intestine of rodents, at birth, there is a maximum expression in the proximal duodenum and gradually decreases in the distal bowel. In rodents, similar to other species where the main transfer of immunity takes place during lactation, this takes place, maternal IgG uptake is low at birth but reaches its highest levels around 14 days of age. At weaning, the absorption of antibodies ceases almost entirely (221, 222). Other studies about Bactrian camels indicate that FcRn was expressed in epithelial cells of the mucosa of the area of aggregated lymphoid nodules (ALNA) of the abomasum of fetuses (10–13 months of gestation), young (1–2 years), pubertal (3–5 years), middle-aged (6–16 years) and older (17–20 years). However, the expression level decreased rapidly in the elderly group. With changes after ALNA matured, the expression level of FcRn in the non-follicle-associated epithelium (non-FAE) was significantly higher than in FAE. FcRn was also detected in vessel endothelium, smooth muscle tissue, macrophages, and dendritic cells of secondary lymphoid follicles (286). However, cell type-specific levels are not known to change with age. It is believed that this would happen and would be influenced by other factors of exposure, endocrine, and immune status. Although there is some expression data in different species and at different stages of life, there are no studies in all of them.

## 7 Conclusions

FcRn belongs to the Fcγ receptors family and is the most recent receptor discovery. FcRn has taken significant importance in the last years because it has been used for multiple applications, mainly medical, for the prevention or treatment of different diseases, due to the receptor is capable of transporting various molecules, whether modified or bound IgG or albumin that could have specific biological activity. All FcγR receptors bind to the Fc fraction of IgG antibodies at different amino acids and trigger different intra- and extracellular processes. The location of the FcγRs in each cell type varies, as well as its activity and the processes it triggers, both intracellular and extracellular. FcRn has certain peculiarities, for example, that it can bind two molecules simultaneously, IgG and albumin, with different stoichiometry, 2:1 and 1:1 ratios, respectively; moreover, at specific acid pH (6.5) and not neutral. These characteristics are different to the other receptors, which bind to IgG at a 1:1 ratio and only at 7.2-7.4 pH.

Furthermore, FcRn can carry out different functions depending on the cell type and the tissue or organ in which it is found. In some cases, it can only remain on one side and inside the cells to recycle the molecules to which it binds. In others, it can pass through the cells to deliver IgG, either monomeric or in the form of immune complexes, and this transport, in general, favors immunological surveillance or the development of a specific response against the antigen-bound and transported together with its antibody. These differences by cell type have led to the hypothesis that FcRn has different kinds of regulation, probably at a post-translational level. Interestingly, according to the comparisons made at the gene, cDNA, and amino acid sequence level, a certain degree of homology is maintained. Despite the changes, the function is kept in the different species analyzed. For these reasons, the intracellular mechanisms generated in each cell type remain to be explored, which depend on intracellular signaling. Once the ligand binds, and what happens when two kinds of molecules bind to the same receptor is of greater interest, it would be believed that the molecule with higher affinity, in this case IgG, would have a more significant influence. Another aspect that is unknown in most cell types is how FcRn expression changes depending on the age of the individuals. It is essential to know the biology of the receptor because this would help expand knowledge about various aspects of the immune system, regulation of IgG and albumin levels, intracellular regulation mechanisms, and their proper maintenance.

## Footnotes

1
https://www.ncbi.nlm.nih.gov/gene.

2
https://useast.ensembl.org/index.html.

3
https://www.uniprot.org/.

## Appendix

The sequences of the FcRn α-chain of each mammalian species analyzed in this paper were obtained to compare characteristics at the gene, mRNA, and protein level, including length, number of exons and introns, changes in the protein domains, binding amino acids to its ligands (IgG Fc, albumin), to β2m, and other key molecules to carry out its FcRn functions, among others. The sequences were downloaded from the Ensemble^1^ and Uniprot^2^ databases, and multiple alignments were carried out to compare them and to obtain phylogenetic trees using the Clustal Omega server. For each species analyzed, the type of placentation it presents was determined. It was observed that at the cDNA and protein levels, the species are grouped according to the type of placenta (**Figures 3**, **4**).

Access numbers from Ensemble database: ENSOCUT00000020921.2: rabbit; ENSMUST00000003512.8: C57BL6 mouse; ENSRNOT00000027944.6: rat; ENSMMUT00000091087.1: macaque; ENSPPYT00000011920.2: orangutan; ENST00000426395.7: human; ENSPTRT00000111176.1: chimpanzee; ENSPPAT00000064950.1: bonobo; ENSGGOT00000008813.3: gorilla; ENSOGAT00000025884.1: bushbaby; ENSFCAT00000051684.1: cat; ENSCAFT00000005836.4: dog; ENSPCTT00005022358.1: sperm whale; ENSBTAT00000031878.2: cow; ENSCHIT00000037360.1: goat; ENSOART00000014354.1: sheep; ENSSSCT00000003540.4: pig; ENSCWAT00000008910.1: peccary; ENSECAT00000003823.2: Horse.

Access numbers from UniProt database: rabbit: ENSOCUT00000020921; C57BL6 mouse: ENSMUSP00000003512; rat: ENSRNOP00000027944; macaque: ENSMMUP00000078324; orangutan: ENSPPYP00000011477; human: ENSP00000221466; chimpanzee: ENSPTRP00000084670; bonobo: ENSPPAP00000042021; gorilla: ENSGGOP00000008578; bushbaby: ENSOGAP00000016161; cat: ENSFCAP00000041331; dog: ENSCAFP00000005404; sperm whale: ENSPCTP00005020300; cow: ENSBTAP00000031824; goat: ENSCHIP00000029490; sheep: ENSOARP00000014147; pig: ENSSSCP00000053999; peccary: ENSCWAP00000008183; horse: ENSECAP00000002663.
